# Assembling Biocompatible Polymers on Gold Nanoparticles: Toward a Rational Design of Particle Shape by Molecular Dynamics

**DOI:** 10.1021/acsomega.2c05218

**Published:** 2022-11-10

**Authors:** Roberta Cappabianca, Paolo De Angelis, Annalisa Cardellini, Eliodoro Chiavazzo, Pietro Asinari

**Affiliations:** † Department of Energy “Galileo Ferraris”, Politecnico di Torino, Corso Duca Degli Abruzzi 24, 10129Torino, Italy; ‡ Istituto Nazionale di Ricerca Metrologica, Strada Delle Cacce 91, 10135Torino, Italy

## Abstract

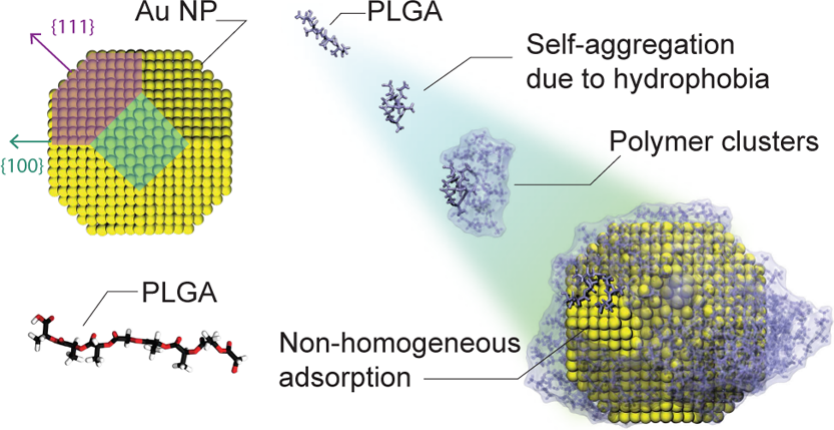

Gold nanoparticles (AuNPs) have received great attention in a number of fields ranging from the energy sector to biomedical applications. As far as the latter is concerned, due to rapid renal clearance and a short lifetime in blood, AuNPs are often encapsulated in a poly(lactic-*co*-glycolic acid) (PLGA) matrix owing to its biocompatibility and biodegradability. A better understanding of the PLGA polymers on the AuNP surface is crucial to improve and optimize the above encapsulation process. In this study, we combine a number of computational approaches to explore the adsorption mechanisms of PLGA oligomers on a Au crystalline NP and to rationalize the PLGA coating process toward a more efficient design of the NP shape. Atomistic simulations supported by a recently developed unsupervised machine learning scheme show the temporal evolution and behavior of PLGA clusterization by tuning the oligomer concentration in aqueous solutions. Then, a detailed surface coverage analysis coupled with free energy landscape calculations sheds light on the anisotropic nature of PLGA adsorption onto the AuNP. Our results prove that the NP shape and topology may address and privilege specific sites of adsorption, such as the Au {1 1 1} crystal planes in selected NP samples. The modeling-based investigation suggested in this article offers a solid platform to guide the design of coated NPs.

## Introduction

Gold nanoparticles (AuNPs) are the subject of intense research, thanks to their optical, electronic, and molecular recognition properties, thereby resulting in particular interest in several applications ranging from energy^1,2^ to medicine.^3^ In the biomedical field, AuNPs have received a large emphasis in cell tracking,^4^ drug delivery,^5,6^ photothermal therapy,^7^ and molecular imaging.^8^ However, the use of AuNPs in these applications often encounters biocompatibility issues. The encapsulation of AuNPs in a biodegradable poly(lactic-*co*-glycolic acid) (PLGA) matrix is a common strategy to preserve the optical and electronic properties of the AuNPs and to improve their biodistribution.^9−11^ To optimize the encapsulation, a precoating of AuNPs with PLGA polymers has often been adopted. A number of research groups have actively investigated how to produce PLGA-coated AuNPs in a controlled manner, principally for their significant potential in cancer treatment therapies.^12^ For example, Xi et al.^13^ have shown that combining the properties of AuNPs with those of PLGA polymers is useful for enabling efficient ultrasound-guided high-intensity-focused-ultrasound techniques for tumor therapy. Alkilany et al.^14^ have described a procedure to guide the efficient encapsulation of AuNPs into PLGA NPs, without using surfactant molecules to assure stability. Their protocol consists in functionalizing the AuNPs with PLGA brushes that easily dissolve and solubilize in the PLGA host matrix, according to the rule of like dissolves like. A number of further experimental approaches have been also tested to design PLGA-coated AuNPs and the evaluation of their impact in a biological environment.^15,16^ Nevertheless, a more theoretical understanding of the PLGA polymer self-assemblies and their adsorption on gold NPs would represent a fundamental step to enhance the efficacy of the NP design and loading in the PLGA matrix for several biomedical applications. Specifically, improving the NP rational design means determining the most favorable surface shape for adsorption.

Recently, many theoretical and computational approaches regarding the interactions between soft materials and inorganic NPs have shown promising advances in the NP engineering protocols, thereby providing a key direction to rationally guide the experimental preparation of Au nanocolloidal suspensions.^17−19^ For example, Tavanti et al.^20^ carried out molecular dynamics (MD) simulations to improve the understanding of the protein–AuNP interactions at the molecular level and to provide suggestions for resolving the uncertain picture obtained by comparing different experimental data. Precisely, they investigated the interaction between three proteins (myoglobin, hemoglobin, and trypsin) and a 15 nm-diameter citrate-capped AuNP. In this study, they determined both the driving forces for the biocorona formation and the protein competition for AuNP binding, showing how the protein shape affects adsorption. In contrast, Tandiana et al.^21^ used a density functional theory (DFT) framework followed by a thorough quantum chemical topological analysis to characterize the interaction between Au and several organic molecules. Specifically, they investigated the adsorption on the AuNP surface of small organic molecules, such as methane, ammonia, and methanol to highlight which are the most reactive sites of the Au. Recent computational works have demonstrated that not only the physicochemical properties of the AuNP surface impact the molecule adsorption at solid–water interface but the NP size and surface morphology may also play a relevant role.^22,23^ In this direction, Perfilieva et al.^24^ have introduced a polarizable force field to describe the interactions between a truncated octahedron AuNP with sodium citrate. Salassi et al.^25^ highlight how different shapes and sizes of AuNPs lead to their different kinds of wrapping with organic ligands. Not surprisingly, other recent works have shown evident effects of the NP surface topology on specific polymer adsorption.^26−28^

In this paper, we combine several molecular modeling approaches to investigate how the particle shape and orientation affect the adsorption mechanisms of PLGA oligomers onto a bare crystalline AuNP. In particular, we carry out atomistic MD simulations of a AuNP and PLGAs in an aqueous environment. By using a machine learning (ML)-based algorithm, we identify the time evolution of PLGA clusters formed at different concentrations. In addition, the analysis of the AuNP solvent-accessible surface after the adsorption elucidates the anisotropic nature of both PLGA self-aggregation and their consequent adsorption on the AuNP surface. By coupling the previous results with a free-energy-landscape (FEL) protocol, we confirm the dominant anisotropy in the formation of a PLGA–AuNP assembly which reveals preferential sites of adsorption according to the NP crystalline structures.

## Methods

### Atomistic Model of Bare AuNPs and PLGA

**Figure 1:**
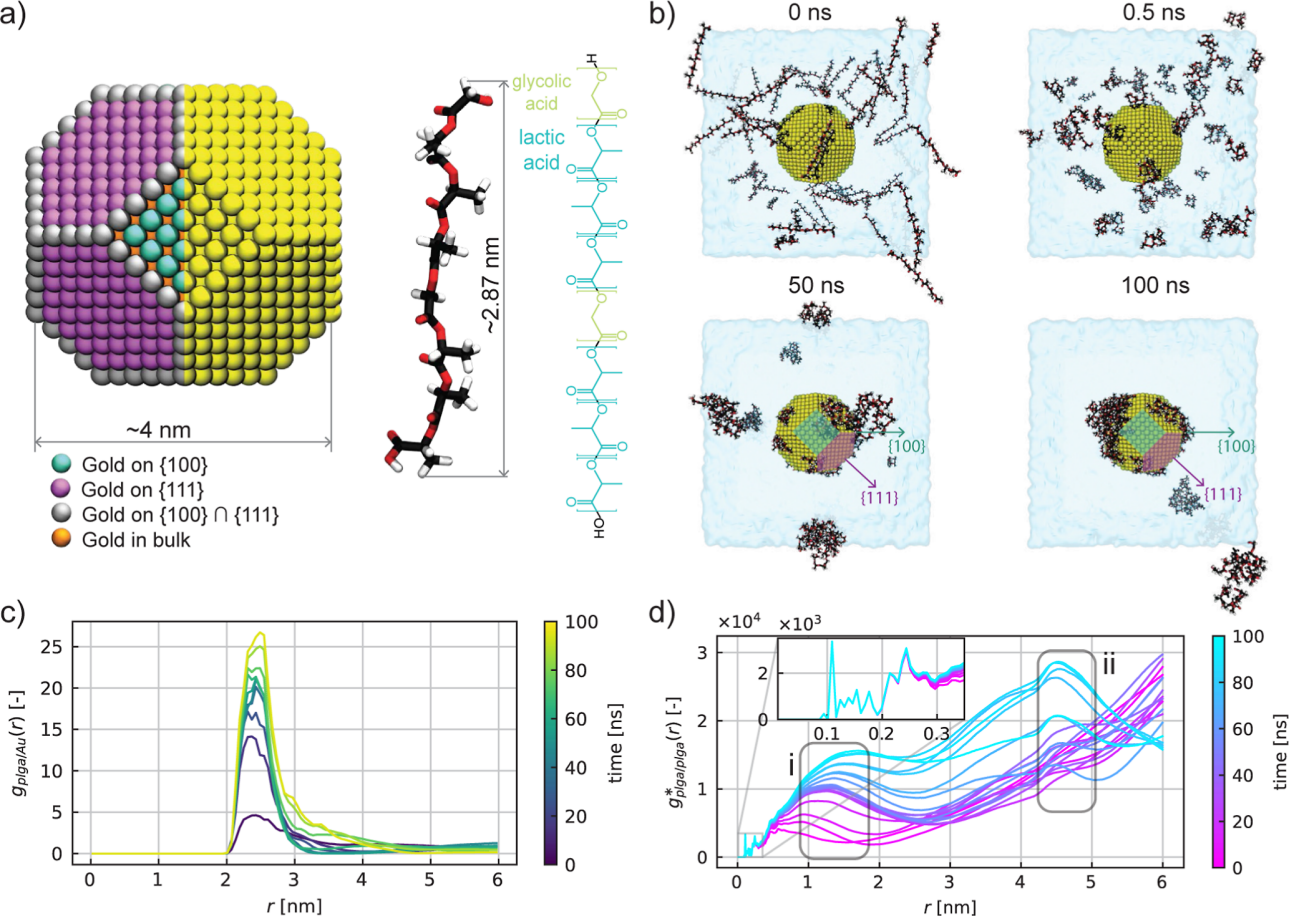
(a) Atomistic representation of the bare crystalline AuNP and PLGA oligomer. The short PLGA chain is made of eight monomers: six LA (light green) and two GA (cyan) residuals (the PLGA topology file, collected in the data repository, reports the molecular residues where the subscript t indicates the terminal groups). Color code for the AuNP: yellow, the total AuNP; orange, Au atoms in bulk; gray, Au atoms on both surfaces {1 0 0} and {1 1 1}; purple, Au atoms belonging to the crystalline plane {1 1 1}; cyan, Au atoms belonging to the crystalline plane {1 0 0}; white, hydrogen atoms; red, oxygen atoms; black, carbon atoms. (b) MD snapshots of self-assembly simulations of 60 PLGAs and the AuNP in aqueous solution at 0, 0.5, 50, and 100 ns. (c) Time evolution of the radial distribution function (*g*_plga/Au_(*r*)) of PLGAs around the center of mass of the AuNP. (d) Distribution of mutual distances between the PLGA atoms  as a function of time; the highlighted peaks i and ii show the evolution of PLGA self-assembly and PLGA adsorption on the AuNP, respectively.

The procedure to define the geometry and topology of a bare AuNP having a diameter of 4 nm was written in the Python language,^29^ and the relative Jupyter Notebook^30^ is available in the Supporting Information. Specifically, we used the Python library atomic simulation environment (ASE),^31^ which includes tools and modules for setting up, manipulating, running, visualizing, and analyzing atomistic simulations. Through one of the ASE modules designed for cluster creation, the gold face-centered cubic unit cell was first replicated along the three Cartesian axes and then cut along the {1 0 0} and {1 1 1} crystalline planes. To create the cluster of desired size and shape, we employed the recursive Wulff construction method.^32−34^ It consists of determining the equilibrium shape of a crystal with a known mass by minimizing the surface energy. Thus, given the equivalent mass of a spherical AuNP with a 4 nm diameter and the ratio between the surface tension of the two crystalline planes, namely, γ_SV_^{111}^/γ_SV_^{100}^ = 0.96,^35^ the algorithm found a NP of 1925 gold atoms with an equivalent diameter of 3.96 nm (Figure 1a). It is worth noticing that the ratio of surface free energy, that is, 0.96, fixed to build the AuNP, is consistent and reliable with the experimental values, as demonstrated by Heinz et al.,^35^ whose force field has also been adopted in this article to model the AuNP. Thus, the resulting AuNP is the reference case studied in this article. However, to show the effect of NP topology on PLGA adsorption, we also arbitrarily varied the AuNP surface shape by modifying the surface tension ratio within the Wulff construction method. In particular, we obtained the NP_A_ by fixing γ_SV_^{111}^/γ_SV_^{100}^ = 0.7 and the NP_C_ by setting up γ_SV_^{111}^/γ_SV_^{100}^ = 1.4 (see Figure 6a). A different protocol to design an amorphous AuNP model is instead described in the Supporting Information.

On the other hand, we simulated small oligomer chains as block copolymers of PLGA made up of eight monomers each: six repeating units of lactic acids (LAs) and two glycolic acids (GAs), as shown in Figure 1a. The chain length of PLGA used in our computational study is much less than that one generally employed in experimental works. Therefore, we refer to our PLGA polymers as small oligomer chains. However, the current investigation is mainly focused on the fundamental mechanisms driving the adsorption phenomenon of single PLGA and PLGA NPs, rather than reproducing the behavior of a specific polymer chain. In addition, we specify that the polymers with a higher LA moiety require more time to degrade by hydrolysis,^36^ so to achieve a good balance between the durability and hydrophilicity, we consider an oligomer with a LA/GA ratio equal to 75:25 (i.e., 566 g/mol molecular weight).

### MD Simulations of PLGA-Coated AuNPs

In order to simulate the self-assembly of PLGAs on the designed AuNP in an aqueous solution, MD simulations were carried out using the open-source software GROMACS 2019.1.^37,38^ We considered four molecular modeling setups including one AuNP with a specific concentration of PLGA, namely, 10, 20, 30, and 60 oligomers chains corresponding to 7.5, 15.1, 22.5, and 45 mM, respectively. Although such concentrations are above the typical values used in bioexperiments,^39^ highly concentrated solutions are generally adopted in MD studies to allow clear observation of the nanoscale phenomena in a reasonable time compatible with the current MD performance. The simulation box was designed with a volume of 13 × 13 × 13 nm^3^ to ensure compliance with the minimum image convention. However, a size-independent study of the results is reported in the Supporting Information where we vary the simulation box size at fixed PLGA concentrations. The Packmol tool^40^ was used to arrange the AuNP in the center of the box and to randomly distribute the PLGA molecules in the rest of the volume. Then, box solvation with water described by the SPC/E model^41^ was performed and periodic boundary conditions were applied in all box directions. The OPLS–AA force field^42^ was used to describe the PLGA oligomer, and we adjusted the PLA dihedral parameters to reproduce both the DFT and the experimental data as implemented by McAliley et al.^43^ In addition, we simulated the PLGA oligomer in its protonated state, that is, with acid caps. Although in a physiological environment, PLGA tends to leave its ester hydrogens and to be in its deprotonated state, more soluble in water, such a deprotonation state slows down the PLGA self-assembly and the consequent adsorption process to the AuNP. Thus, to accelerate the adsorption of PLGA on the AuNP surface, we modeled the PLGA oligomer in its protonated state and we demonstrated that the presence of OH groups only affects the velocity of the aggregation mechanisms, leaving the main findings of this work unchanged (see Figure S10 in the Supporting Information for further details). On the other hand, the AuNP was modeled by using the nonbonded parameters validated by Heinz et al.^35^ to obtain the experimental surface free energy and interface properties with water and (bio)organic molecules. All simulations were carried out by employing the LINCS algorithm to restrain the covalent bonds involving hydrogen atoms. We point out that the adsorption of PLGAs on the AuNP surface is only driven by nonbonded interactions, namely, the integration of van der Waals and Coulomb potentials between all atoms belonging to Au and PLGA, respectively. Because the effects of partial charges are neglected in our Au model,^35^ then, the net Au–PLGA interaction energy is given by the superposition of 12-6 Lennard–Jones (LJ) potentials.

Both van der Waals and short-range electrostatic interactions were evaluated within a cutoff radius of 1 nm, while for the remaining long-range interactions, a particle-mesh Ewald^44^ summation was applied to resolve electrostatics in the Fourier space. Our MD protocol consists of the first step of energy minimization and two consequent equilibration steps. Initially, to reach an equilibrium temperature of 300 K, we applied the canonical ensemble (*NVT*) for 100 ps using a Maxwell–Boltzmann speed distribution and the *V*-rescale thermostat^45^ with a time constant τ_*t*_ = 0.1 ps. Subsequently, we set the isothermal-isobaric (*NPT*) ensemble for 100 ps at an equilibrium pressure of 1 bar and an equilibrium temperature of 300 K. In this step, we used the Parrinello–Rahman barostat^46^ with a time constant τ_*p*_ = 2 ps and the *V*-rescale thermostat with a time constant τ_*t*_ = 0.1 ps. During the equilibration steps, both the oligomer chains and the AuNP were restrained in their initial positions using a harmonic potential with a force constant of 1000 kJ mol^–1^ nm^–2^. Once the desired thermodynamic conditions were reached, the restraining on the small polymer chains was removed and the self-assembly process of the PLGAs was observed for the next 100 ns. It is worth noticing that this simulation time has revealed a reasonable timescale to observe and capture the peculiar phenomena linked to PLGA adsorption. Configurations with different concentrations of PLGA were simulated following the illustrated protocol. All MD simulations integrate Newton’s second law with the Leap-Frog^47^ algorithm and a time step of 2 fs.

### Unsupervised Learning to Explore PLGA Clusters

**Figure 2:**
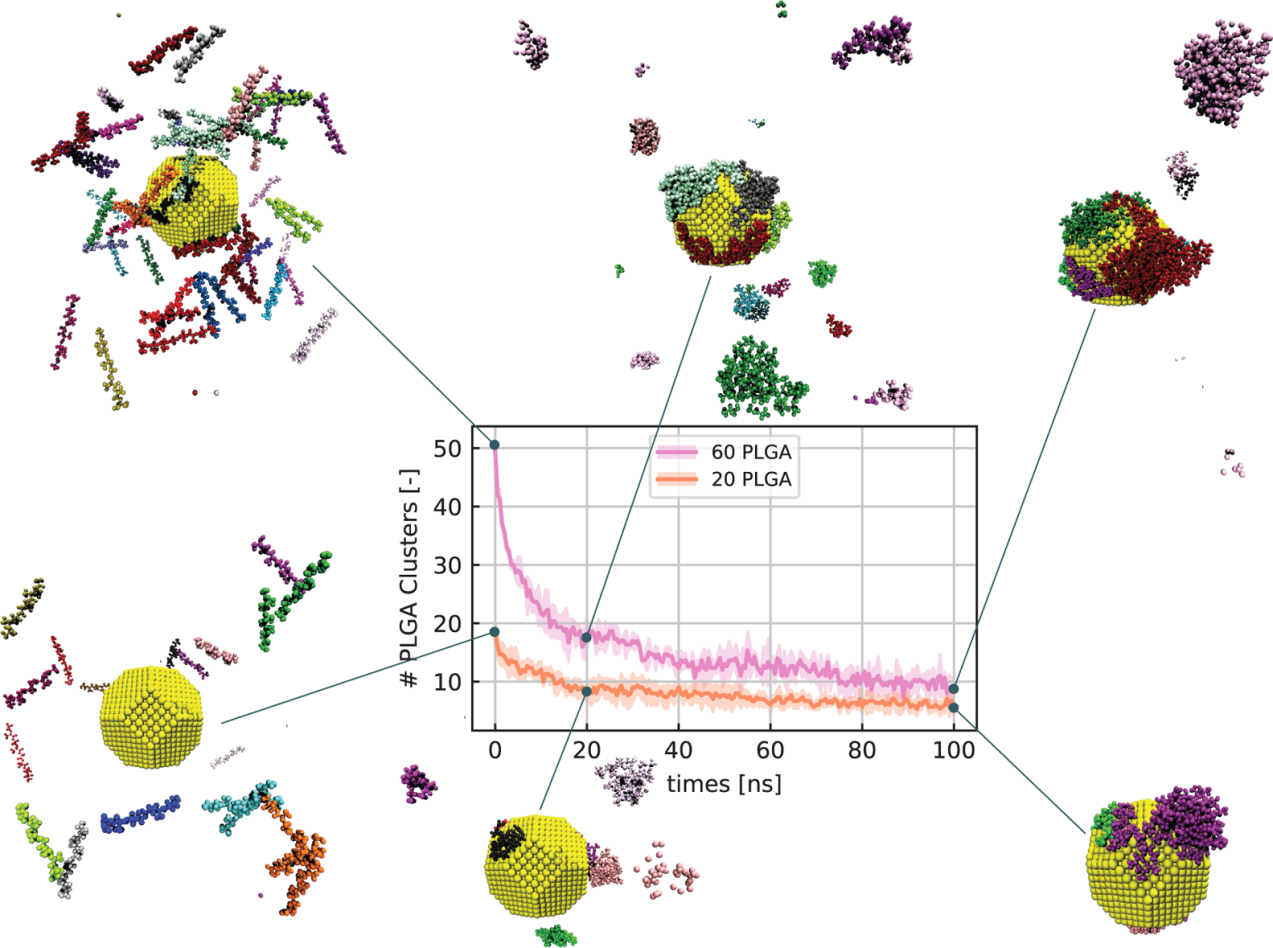
Number of PLGA clusters formed during 100 ns of MD simulation in case of 60 initial PLGA molecules (pink line, top snapshots) and 20 initial PLGA molecules (orange line, bottom snapshots). The color code in the MD snapshots shows the atoms belonging to different clusters (note that yellow is used to identify the AuNP). The number of clusters is obtained by averaging the results over three replica copies of the same MD simulation, and the shaded region represents the standard deviation error.

Taking advantage of the formidable ability of ML in terms of feature extraction^48,49^ and cluster analysis, we applied an unsupervised learning scheme to investigate the time evolution of self-assembly pathways of the oligomer chains. We carried out a clustering analysis by adopting the algorithm of Adorf et al.^50^ used for crystallization events to our PLGA coating phenomena. Specifically, the HDBSCAN* algorithm,^51^ which is an evolution of the well-known DBSCAN,^52,53^ was applied and the extension of the open-source scikit-learn library^54,55^ was added. In general, the goal of a clustering study is to classify objects of a database into a set of meaningful subclasses. The added value of HDBSCAN* is to allow clusters of varying density to be found and to be robust against fluctuations in density and cluster size. The algorithm identifies clusters as high-density areas separated by low-density areas; as a consequence, the clusters found can be of any shape. The hyperparameters are the cluster selection mode and the metric, that are, *leaf* and manhattan, respectively. In addition, there are two further hyperparameters: (i) *min_samples*, which mainly controls the tolerance of the algorithm toward the noise, and (ii) *min_cluster_size*, which is the minimum size to define a cluster. Ideally, the latter hyperparameter is relatively intuitive to select: set it to 69, that is, the number of atoms forming one PLGA which is the smallest cluster size in our application. However, it is necessary to find an optimal condition between the parameters *min_cluster_size* and *min_samples* to avoid some PLGAs being considered noise. Therefore, we varied the two hyperparameters to reach the optimal condition where the initial number of clusters is approximately equal to the number of PLGAs in the solution. Coherently with our application, we set *min_samples* = 7 and *min_cluster_size* = 60. The same hyperparameters were used to study the self-assembly pathways of the different configurations under investigation (Figure 2).

### Umbrella Sampling Approach to Compare FELs

We used the umbrella sampling (US) approach for the calculation of the potential of mean force (PMF) related to the adsorption of a single PLGA oligomer chain on the AuNP. Using the Packmol routine, we created the initial configurations in which the PLGA is placed as close as possible to the AuNP surface. After performing few steps of energy minimization, we achieved the equilibrium state of PLGA adsorption: no additional attraction is expected at a shorter distance. The box has a volume of 7 × 7 × 13 nm^3^, which is sufficient to allow continuous traction along the *z*-axis without being affected by the periodic imaging of the system. The AuNP was placed at *x* = 3.5 nm, *y* = 3.5 nm, and *z* = 3.5 nm, and we solvated the box with the SPC/E water model. We minimized the energy of the system and equilibrated the box as previously described in our MD protocol. Once the thermodynamic conditions were reached, a 2.5 ns MD simulation was carried out, in which the NP was constrained to its initial position, while the PLGA was pulled along the *z*-axis at a rate of 1 nm per nanosecond. Saving snapshots every 1 ps, the reaction coordinate (ξ = *z*) was divided into single sampling windows. We chose enough sampling windows to achieve a regular spacing and performed additional MD simulations for each window. The center of mass of the PLGA was constrained in each window by an umbrella harmonic potential with a force constant of 10,000 kJ mol^–1^ nm^–2^. For each selected window, we applied our MD protocol starting from the equilibration step in the *NPT* ensemble and concluding with 10 ns of the production run. The simulation time was chosen to ensure a good sampling and hence a good statistical analysis, as confirmed by the convergence process shown in Figure S6 in the Supporting Information. We finally obtained the PMF profile using the weighted histogram analysis method^56^ included in GROMACS (see the umbrella histograms in Figure S5 of the Supporting Information). The umbrella protocol was repeated four times by modifying the angular positions of the AuNP and the PLGA oligomer (Figure 4).

## Results and Discussion

### PLGA Self-Assembly and Adsorption on the AuNP

MD simulations were carried out to study the self-assembly and adsorption phenomena of PLGA oligomers onto a AuNP surface. In order to observe and understand how PLGAs coat the NP in the early stage of adsorption, we used extensive simulations of a tunable concentration of PLGA oligomer chains in an aqueous environment. Specifically, we dealt with four distinct case studies consisting of a single AuNP and 60, 30, 20, and 10 PLGA chains, respectively, randomly placed in a box of 2197 nm^3^. Atomistic representations of the considered NP and oligomer chain models are shown in Figure 1a, where the {1 0 0} and {1 1 1} planes are identified in cyan and purple colors, respectively. Figure 1b, instead, shows four representative snapshots of the oligomer-NP self-assembly process in case of the most concentrated solution, namely, 60 PLGA and one single AuNP at 0, 0.5, 50, and 100 ns. Note that some of the representative MD snapshots for less concentrated PLGA solutions are reported in Figure S2 of the Supporting Information. From a qualitative point of view, the snapshots first display the self-aggregation of PLGA chains into small polymeric particles (see Figure 1b, at 0 and 0.5 ns), thereby confirming the polymer’s hydrophobic nature.^57,58^ Subsequently, the assembled PLGAs tend to be adsorbed on the AuNP surface (see Figure 1b at 50 and 100 ns). A more quantitative validation of the progressive PLGA adsorption on the AuNP surface is demonstrated in Figures 1c and S3 by the radial distribution functions, *g*(*r*), of the PLGAs from the center of mass of the gold NP. The *g*(*r*) peaks are observed at a distance (*r*) of about 2.5 nm, confirming the presence of adsorbed oligomers on the AuNP surface (note that the AuNP radius is roughly 2 nm as pointed out in Figure 1a). Moreover, Figure 1c also shows that the distribution of the PLGA chains adsorbed on the gold surface enhances as the simulation time increases. To further investigate the PLGA reconfiguration after the aggregation processes, we analyzed the relative distribution of the atoms within the PLGA molecules. Explicitly, we calculated the PLGA atomic distribution , without normalizing it over the averaged PLGA atomic density (see Figure 1d). First, we note that the trend of *g*_plga/plga_^*^(*r*) for *r* smaller than approximately 0.3 nm coincides with the *g*(*r*) of the PLGA atoms within a single molecule; in other words, it describes the oligomer structure which does not depend on the evolution of the self-assembly simulation, but it remains unchanged in time (zoom in Figure 1d). Second, we observe that during the initial 40 ns of MD simulations, the *g*_plga/plga_^*^(*r*) grows almost linearly with distance, confirming a homogeneously dispersed solution of oligomers. However, close to 100 ns of simulation, two distinct peaks are notable, the first one between 1 and 2 nm and the second one at about 4.5 nm. While the former indicates that the PLGAs tend to self-aggregate and form larger and larger polymeric clusters, the latter is mostly related to the distribution of PLGAs on the surface of the AuNP, which has a diameter of roughly 4 nm.

Therefore, the self-assembly simulations together with the radial distribution analysis reveal that the PLGA oligomer chains are subject to two distinct and successive aggregation mechanisms: (i) self-assembly into small polymeric soft particles and (ii) the adsorption of small PLGA particles on the AuNP surface.

Once we clarified the aggregation mechanisms in the aqueous environment, we carried out a more detailed analysis using an unsupervised ML technique allowing us to identify and distinguish the number of PLGA clusters formed in the aqueous solution. Specifically, we computed a clustering analysis on the four case studies through the HDBSCAN*^51^ algorithm, which is an evolution of the well-known DBSCAN.^52,53^ This algorithm identifies clusters as high-density areas separated by low-density areas. As a result, clusters can be defined as groups of atoms of any shape but with a minimum size of 60. Figure 2 displays the results obtained for the two extreme case studies, namely, at 60 PLGA and at 20 PLGA concentrations, while the self-assembly phenomena of 30 and 10 PLGA in solution are reported in Figure S4 of the Supporting Information. For each concentration considered, we performed three independent self-assembly simulations of PLGAs and we report the mean and standard deviation of the time evolution of clusters by averaging the results over the three MD-independent replicas. The outcomes in Figures 2 and S4 reveal that the PLGA self-assembly is faster than the adsorption on the AuNP surface and occurs mainly during the first 20 ns of simulation. Furthermore, comparing the systems with 20 and 60 PLGA, we observe a faster polymer self-aggregation process as the concentration increases. Finally, the snapshots of the coated AuNPs at the final simulation step (100 ns) qualitatively show that the PLGAs cover the AuNP in a nonhomogeneous manner, leaving a percentage of the AuNP surface still bare. These outcomes are also confirmed by including some chemical variations in the description of both PLGA and AuNP models. In particular, Figure S9 in the Supporting Information document demonstrates that a polarizable force field for gold atoms^59^ does not affect the PLGA self-assembly in clusters but weakly slows down their adsorption on the AuNP surface, which results in less coating due to the enhanced hydration of the AuNP. In other words, the higher hydration energy of the gold interfaces resulting from the polarizable model (see the work of Geada et al.^59^) determines a more stable hydration shell around the nanoparticle which tends to slow down the adsorption dynamics of PLGA. Similarly, although the effect of PLGA deprotonation (Figure S10 in the Supporting Information) lowers the velocity of adsorption because of the increased PLGA hydrophilicity, the aggregation of charged PLGA oligomers on the AuNP also clearly unveil the anisotropy feature of adsorption.

### NP Shape Effect on the Adsorption Mechanism

**Figure 3:**
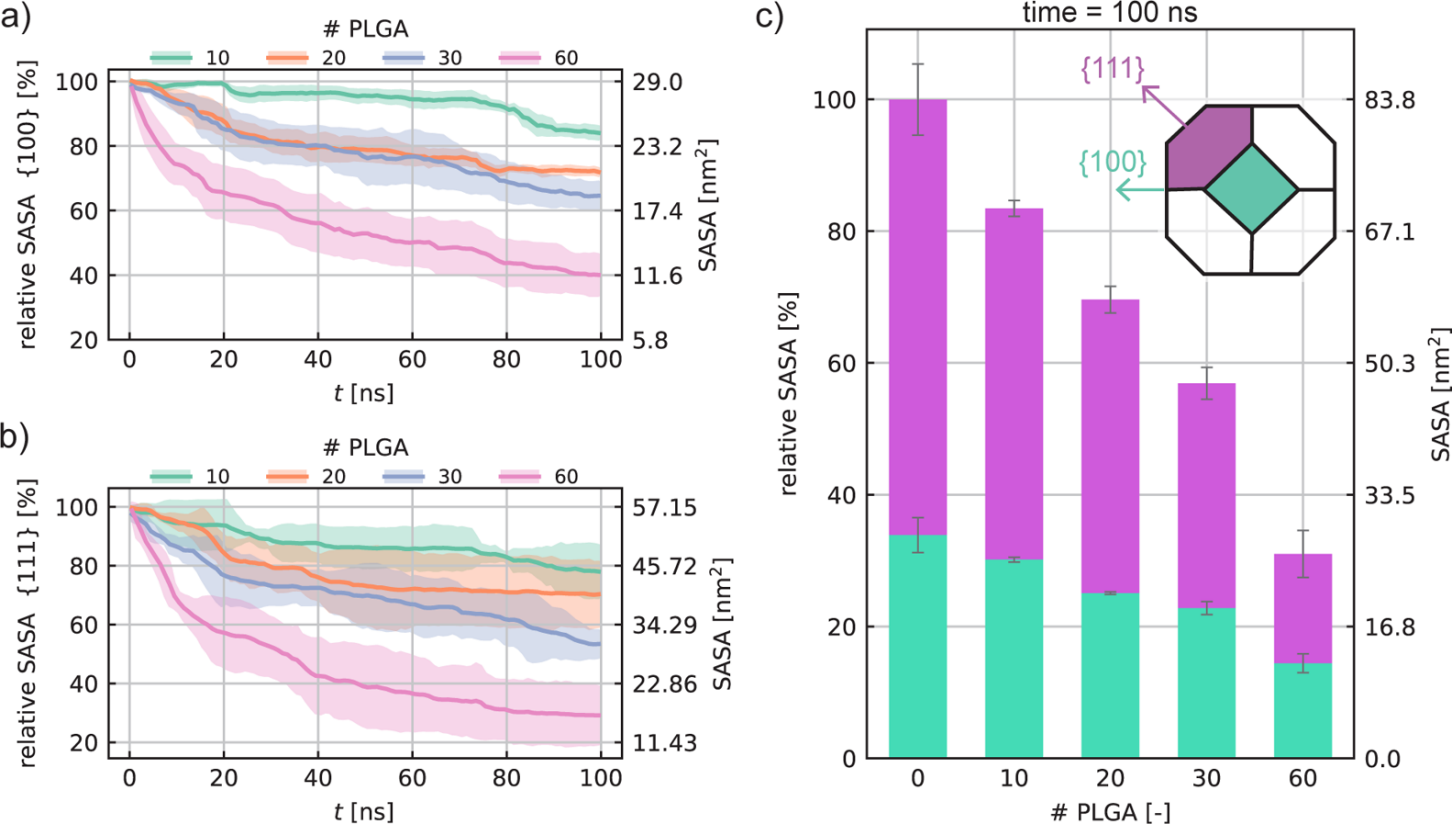
SASA: the SASA trends of {1 0 0} (a) and {1 1 1} (b) crystalline planes at different oligomer concentrations (10, 20, 30, and 60 PLGAs). The relative SASA indicates the percentage values calculated by taking the bare crystalline planes, that is, in the absence of PLGA, as the reference surface area. Different colors illustrate the results obtained for the considered concentrations: green, 10 PLGAs; orange, 20 PLGAs; blue, 30 PLGAs; pink, 60 PLGAs. (c) Histogram of the total AuNP SASA at the last time step simulated, namely, 100 ns, for different PLGA concentrations. Each bar is divided into two colors to distinguish the contribution of the two crystalline planes: purple color for the {1 1 1} plane, and cyan color related to the {1 0 0} plane. Note that the left *y*-axes in (a–c) plots are the percentage values of the relative SASA over the initial surface area, while the right y-axes indicate the absolute value of the solvent-accessible surface of {1 0 0} (a), {1 1 1} (b) planes, and of the total AuNP surface (c). The SASA trends are obtained by averaging the results over three replica copies of the same MD simulation, and the shaded region represents the standard deviation error.

In light of our qualitative results showing the formation of anisotropic PLGA-coated AuNPs, we considered a more thorough investigation of the contribution of NP surface morphology to PLGA adsorption. It is well known that chemistry plays a key role in adsorption phenomena on inorganic NP surfaces.^60−62^ However, more recent studies have shown a peculiar influence of NP topology in the formation of self-assembling supramolecular structures.^25^ To quantify such an effect, we analyzed the four case studies by monitoring the solvent-accessible surface area (SASA) per crystalline plane during the first 100 ns of MD simulations (Figure 3). A reduction of the water-accessible surface has an equivalent meaning of an enhancement of the PLGA adsorption on the specific NP plane. Thus, we evaluated the time evolution of SASA corresponding to the crystalline planes {1 0 0} and {1 1 1} in case of the four PLGA concentrations (see Figure 3a,b). As illustrated in Figure 3a,b, at *t* = 0 ns, the AuNP presents 100% of its surface in contact with water molecules. Along the adsorption process, the percentage of the NP area exposed to water reduces for both {1 0 0} and {1 1 1} planes and in all the tested PLGA concentrated systems. However, some differences arise from this analysis. First, the decrease of SASA due to the PLGA adsorption is more pronounced for higher concentrated systems. The {1 1 1} SASA, for example, reduces by 20, 40, and 73% after 100 ns in solutions with 10, 20, and 60 PLGAs, respectively (green, orange, and pink profiles in Figure 3b). In other words, in higher concentrated systems, the PLGA adsorption on the AuNP occurs faster than in a more dilute solution. Second, comparing Figure 3a,b, the percentage of SASA decreases more rapidly for the {1 1 1} crystalline planes than for the {1 0 0} planes, regardless of the PLGA concentrations. This means that the PLGA oligomer chains preferentially adsorb on the {1 1 1} plane than on the {1 0 0} plane. Clear evidence is shown in case of 60 PLGA: after 40 ns, while the {1 1 1} uncoated surface (i.e., the relative SASA) is 40% of the initial {1 1 1} area, the percentage of bare {1 0 0} planes corresponds to 60% of the total {1 0 0} area. These outcomes quantitatively confirm not only the anisotropic nature of the PLGA adsorption but also give insights into the reason for such asymmetry that is mostly related to the NP crystalline structure and topology. Interestingly, the AuNP polarizable force field and the PLGA deprotonation do not affect the anisotropic behavior of adsorption as shown in Figures S9 and S10 of the Supporting Information, respectively. In Figure 3c, we report the total SASA of the AuNP after 100 ns. The histogram displays the percentage of the total AuNP surface still uncoated and highlights the proportion between the {1 1 1} (purple bars) and {1 0 0} (cyan bars) area exposed to water. In case of 60 PLGA, the percentage of the bare AuNP surface after the adsorption is roughly 34% of the total AuNP area. Such an uncoated surface exposed to water presents a 50:50 ratio between the {1 1 1} and {1 0 0} planes, in contrast to the intrinsic plane surface ratio of the considered AuNP corresponding to 66:34 (see 3c with 0 PLGA). The latter result is a clear demonstration that although the AuNP presents more {1 1 1} than {1 0 0} area, after adsorption, the two planes are equally exposed to water, which implies preferential PLGA adsorption on the {1 1 1} planes. This analysis ultimately confirms that the PLGA-NP coating is strongly influenced by the specific NP crystalline structure, showing a remarkable asymmetry while aggregation takes place, regardless of the specific surface chemistry of the AuNP and PLGA. It is worth noticing that the previous results and in particular the anisotropic nature of PLGA adsorption on AuNP mainly involve the early stages of the adhesion process, that is, the first hundreds of nanoseconds. Therefore, such an outcome should be interpreted as a nanoscale phenomenon. We also point out that the full coating of the AuNP; namely, the decay of SASA to zero would require much longer timescales and simulations at constant chemical potential would be needed.

**Figure 4:**
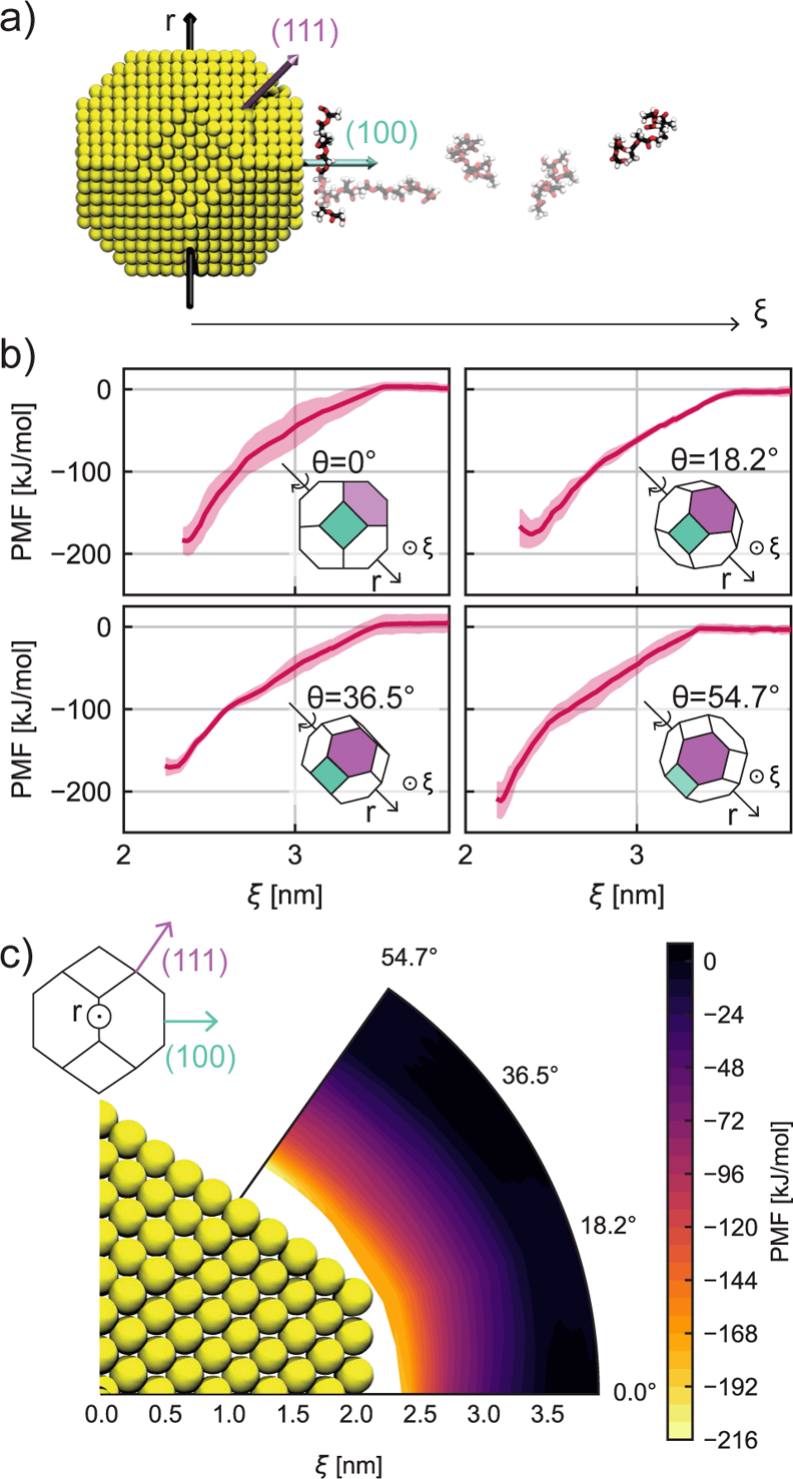
(a) Representative snapshots of the steered MD simulations carried out to pull one single PLGA molecule from the {1 0 0} interface to the bulk solution along the perpendicular reaction coordinate, ξ; the normal vectors to the planes {1 0 0} and {1 1 1} are depicted in cyan and purple arrows, respectively. (b) PMFs obtained by the US method applied to the PLGA and AuNP at four distinct reciprocal orientations; these four reciprocal configurations are realized by rotating the AuNP around the *r*-axis by θ angles. The shaded regions represent the standard deviation of the averaged PMFs over the three US replicas per each reciprocal orientation. (c) Polar coordinate FEL of a AuNP interacting with a PLGA; the polar coordinates used are θ, which is the relative orientation between the AuNP and the PLGA, and the radial distance ξ, which is the reaction coordinate.

In order to lighten the intrinsic nature of such asymmetry and to explain why the nanoscale adsorption also depends on the NP crystalline structure, we explored the FEL using the US method.^37^ In particular, we investigated the FEL of one single PLGA oligomer chain adsorbing on the AuNP by varying their reciprocal angular positions. We rotated the AuNP around the axis *r*, orthogonal to both crystalline plane normal vectors (see the schematic NP sketch in Figure 4a), and we selected four different configurations corresponding to four distinct θ rotation angles (see Figure 4b). While θ = 0° and θ = 54.7° are consistent with the reaction coordinate, ξ, perpendicular to {1 0 0} and {1 1 1} planes, respectively, the other two orientations, θ = 18.2° and θ = 36.5°, are considered to show intermediate cases. Figure 4a schematically represents the spatial AuNP-PLGA arrangements in case of θ = 0°. In line with the US approach, here a PLGA oligomer chain is forced to explore several positions along the reaction coordinate, ξ, which is in this case perpendicular to the {1 0 0} plane. We repeated these simulations three times for each configuration. Figure 4b instead reports the trends of the calculated PMF for the different angular positions of the AuNP. Specifically, we averaged the results over the three replica copies, and in Figure 4b, we show the average trends and standard deviation. In all four orientations, the PMF profile tends to be zero as the distance between AuNP and PLGA increases, while it shows a minimum when the PLGA is adsorbed at the NP–water interface. This confirms that the adsorption process spontaneously occurs. However, as displayed in Figure 4b, the lowest free energy value, Δ*G*, is reached for 2 nm < ξ < 2.5 nm and when θ = 54.7°, namely, when the PLGA is in contact with the {1 1 1} plane, making it the most favorable site of adsorption. On the other hand, the two intermediate angular positions are the most unfavorable sites. Finally, in Figure 4c, the FEL is plotted in polar coordinates. Here, the FEL investigation allows us to confirm the previous considerations: (i) the PLGA does not feel the presence of AuNP further than 1.2 nm from the NP surface (ξ = 2 nm); (ii) the most favorable site for the PLGA adsorption is the crystalline plane {1 1 1} where we observe a deeper FEL well (light-yellow regions).

**Figure 5:**
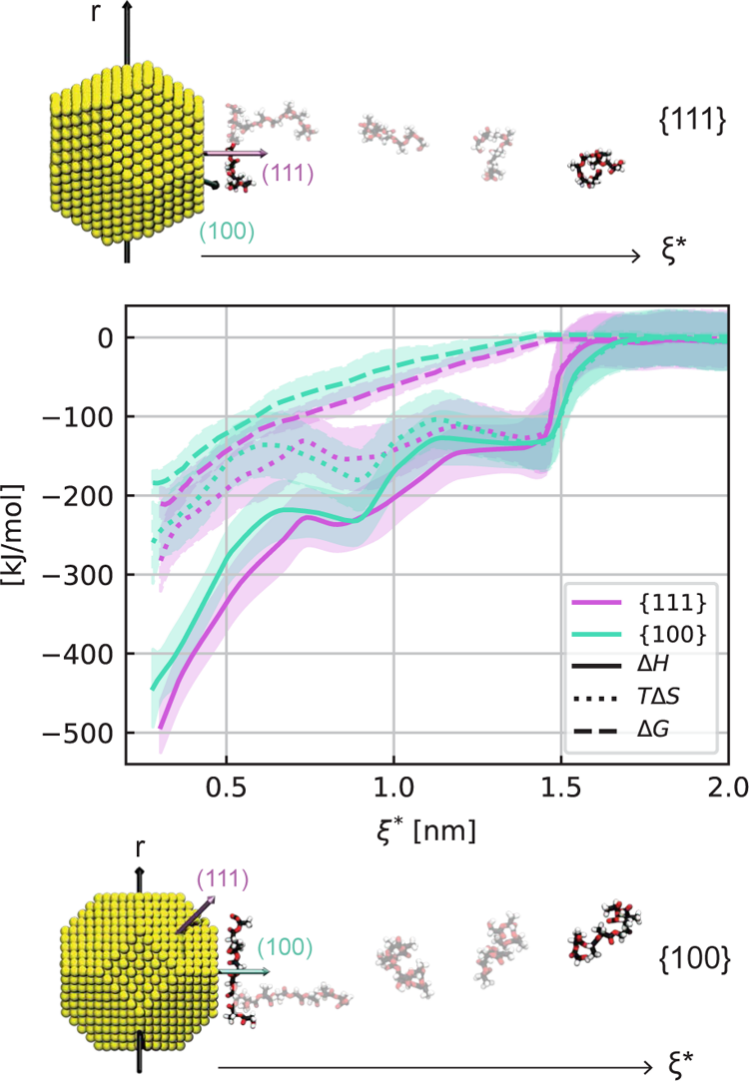
Free energy (Δ*G*—dashed line), entropy (*T*Δ*S*—dotted line), and enthalpy (Δ*H*—solid line) variations during the adsorption of one single molecule of PLGA on the AuNP surface along the reaction coordinate ξ* = ξ – *R*_AuNP_, where *R*_AuNP_ is the AuNP radius. Note that the entropy contribution, *T*Δ*S*, includes both the thermodynamics state function, *S*, and the system temperature, *T*. The plot shows the two extreme NP orientations, i.e., one when the reaction coordinate is orthogonal to the {1 1 1} plane (purple) and the other one when it is orthogonal to the {1 0 0} plane (in cyan). The MD snapshots show some of the US windows in case of PLGA adsorption toward the {1 1 1} (top) and {1 0 0} planes (bottom).

To clarify whether the nature of anisotropic self-assembly on the AuNP can be considered enthalpic or entropic, we estimated the behavior of LJ and Coulomb interactions, while a single PLGA chain approaches the AuNP (Figure 5). In fact, focusing on such an isolated adsorption phenomenon, the variation of the LJ and Coulomb potentials along a specific reaction distance can be related to the change of the enthalpic contribution (Δ*H*) during the aggregation. It is worth noting that the Au–PLGA nonbonded interactions are only driven by the superposition of the 12-6 LJ potential (see the Methods section for further details), while electrostatic forces arise because water molecules also play a role in the enthalpic difference (Δ*H*). On the other hand, the PMF profiles obtained by the US approach represent the free energy change (Δ*G*), bringing a single PLGA molecule from the bulk condition to the AuNP interface. With this in mind and recalling some thermodynamics concepts (Δ*G* = ΔH – *T*Δ*S*), we may estimate the entropic effects during a single adsorption process as a difference between the free energy and the enthalpic variation. Note that the entropy contribution, *T*Δ*S*, includes both the entropy thermodynamics state function, *S*, and the system temperature, *T*. Figure 5 displays the profiles of such thermodynamics state functions along the reaction coordinate, ξ, perpendicular to both {1 1 1} (top sketch and purple lines in the plot) and {1 0 0} (bottom sketch and cyan lines in the plot) planes. We first notice that the entropic contribution arising from Δ*G* – Δ*H* difference increases, in absolute value, approaching the AuNP surface, thereby showing a remarkable impact at the AuNP–water interface. However, the *T*Δ*S* profiles are essentially independent of the specific NP crystalline plane. In other words, the discrepancy in free energy due to the adsorption on {1 1 1} and {1 0 0} planes is maintained constant along the reaction distance and such difference between the planes should be finally interpreted by the PLGA–AuNP noncovalent interactions in water. Therefore, we state that the preferential adsorption on the {1 1 1} has a more enthalpic than entropic character.

**Figure 6:**
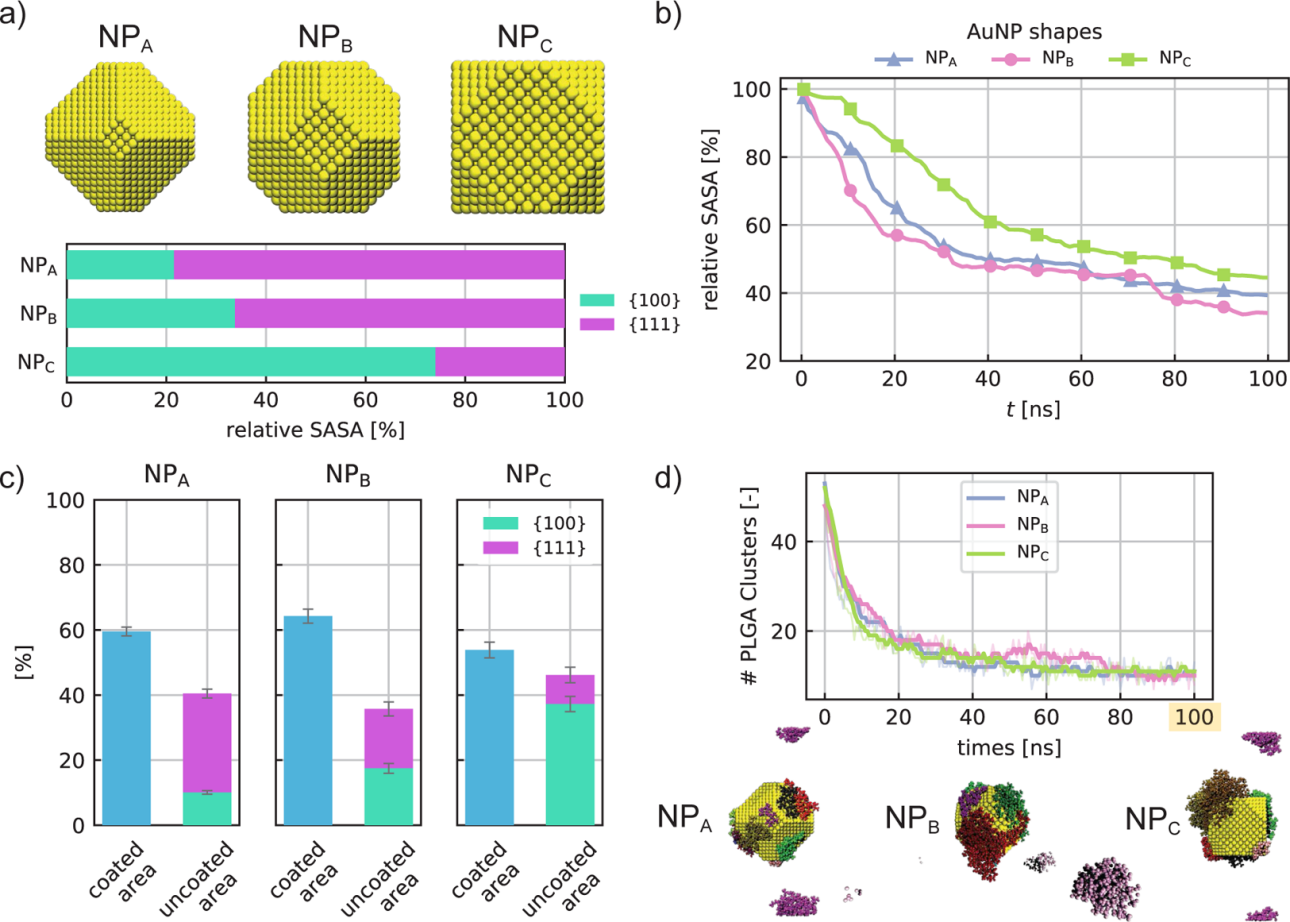
Effect of the NP morphology on the PLGA adsorption phenomenon. (a) Atomistic representation of the considered AuNPs, namely, NP_A_, NP_B_, and NP_C_, which differ from each other for the surface area ratio between {1 0 0} and {1 1 1}; the histogram shows the resulting relative SASA per crystalline plane. (b) The AuNPs PLGA coating dynamics are described by the SASA evolution during the simulated time. Note that the SASA results refer to the adsorption of 60 PLGAs onto the three AuNPs. (c) Percentage of the PLGA-coated area (blue bars) and uncoated area split into {1 0 0} (cyan bars) and {1 1 1} (purple bars) surface planes. (d) Time evolution of the PLGA clusters formed during the self-assembling simulations of 60 PLGAs in case of NP_A_, NP_B_, and NP_C_ in an aqueous solution. The three MD snapshots are the coated NPs in their final configuration at 100 ns (note that the color code used shows the atoms belonging to different clusters).

The results presented so far have unveiled that the peculiar anisotropic coating consequent to the PLGA adsorption is mainly driven by the NP crystalline structure and poorly affected by extra phenomena of entropic nature. In other words, the entropy tends to weaken the spontaneous process of PLGA adsorption, but it is not responsible for specific sites of adsorption. Instead, the greater attraction on the {1 1 1} plane is mostly related, in the absence of specific interactions, to the atomic packing factor (APF) (APF = 0.907 for the {1 1 1} plane and APF = 0.785 for {1 0 0} plane) which influences the number of atoms included within the cutoff radius and consequentially the contributions involved in the Au–PLGA interaction. This observation has suggested the possibility of a more rational design of AuNP shape to improve and enhance the efficacy of the AuNP coating by PLGA oligomers. With this perspective in mind, we carried out a preliminary investigation by designing two further AuNPs and modifying only their intrinsic ratio between the {1 1 1} and {1 0 0} surfaces. Specifically, we built a NP_A_ exhibiting a predominance of {1 1 1} planes, roughly 78% of the total area, and a NP_C_ where the {1 0 0} planes cover 74% of the total AuNP surface area. Figure 6a quantitatively shows the comparison of these extra nanostructures with our standard AuNP (NP_B_), widely studied and already presented in this article. It is worth noticing that such design testing has been carried out to simply validate the effects of NP shape, without any intention of performing a rigorous screening of NP design. Therefore, the ratio between crystal planes was not chosen in a manner consistent with the experimental values, but such as to show the two extreme conditions, that is, the predominance of the {1 1 1} and {1 0 0} planes, respectively. However, we can consider the selected nanostructures reliable with the experimental ones, as in the case of distinct hierarchical crystals with different shape controls obtained by Smith et al.^63^ In order to simply give some indications regarding the AuNP structural influence, we first explored the time evolution of adsorption in case of 60 PLGA oligomers in solutions. The results in Figure 6b show, as expected, a slower PLGA adsorption on the NP_C_, that is, the one with a dominance of {1 0 0} planes. The velocity of PLGA adsorption is instead comparable in case of large {1 1 1} surfaces at the water interface (blue and pink trends in Figure 6b). After 100 ns of PLGA–AuNP self-aggregation, NP_A_ and NP_B_ have a surface-coated area slightly higher than 60% with respect to the total NP surface, while the PLGA coating on NP_C_ is roughly equal to 55.5% (see Figure 6c). This result already confirms that the PLGA adsorption is moderately prevented on AuNPs with a prevalence of {1 0 0} planes. In addition, focusing on the relative SASA of NP_C_ after the adsorption (cyan and purple bars in Figure 6c), the percentage of {1 0 0} plane surface reaches 80% of the total uncoated area, higher than the 74% designed before the adsorption for the same NP_C_. Such preliminary findings first demonstrate the functional role of the NP shape and structure in the adsorption phenomena and, second, the actual prospect of a tailored NP shape design for a more rational PLGA coating. Lastly, Figure 6d emphasizes that the PLGA self-aggregation is weakly influenced by the specific crystalline structure of the AuNP, thereby showing a similar velocity of self-assembling regardless of the type of NP shape. Most probably, the short-range interactions involving PLGA and AuNP affect the adsorption of small PLGA clusters close to the AuNP interface; however, they weakly control the oligomer dynamics at larger distances.

## Conclusions

Coating AuNPs with PLGA polymer chains has gained large potential in biomedical applications. Although several experiments demonstrate the advantages of PLGA coating protocols, relatively few studies have focused attention on the physical-chemistry phenomena controlling the PLGA adsorption on the Au crystalline structure. In this paper, we combined multiple computational approaches to understand how PLGA adsorption can be rationally engineered. The atomistic MD simulations qualitatively demonstrate the adsorption mechanisms of PLGA oligomers on a single AuNP in an aqueous solution. In particular, the time evolution of the radial distribution functions first points out preliminary polymer self-assembly in larger PLGA clusters and, second, their consequent aggregation on the AuNP. This result confirms the validity of our finding regardless of the PLGA polymer length: longer PLGAs may actually change the conformation of polymeric particles formed in water, but they may not affect the key factors driving the interactions with AuNP surface. Moreover, the use of a machine-learning-based algorithm demonstrates and confirms that the rate of PLGA cluster formation is higher in case of 45 mM PLGA concentration than in 15 mM. Beyond the self-assembly analysis, our results provide detailed insights into the anisotropic nature of PLGA coating. A thorough investigation of the AuNP solvent-accessible surface reveals that the kinetic asymmetry regarding the PLGA adsorption is related to the specific crystallographic nature of the AuNP; namely, the AuNP {1 1 1} crystal plane results in a more favorable site of adsorption than the {1 0 0} plane. Moreover, focusing on a single event of adsorption, the coupling of the US technique to the evaluation of LJ interactions, confirms that the anisotropic adsorption has a more enthalpic than entropic nature. Finally, examples of diverse AuNP shapes demonstrate that the adsorption mechanisms can be tuned and controlled not only by the chemistry but also by a rational and functional design of the AuNP topology. In conclusion, our findings reveal that a more efficient PLGA coating and hence a more effective AuNP encapsulation in the PLGA matrix may be addressed with a rational tuning of NPs surface topology. In this direction, current technological advances already provide solid platforms and instruments to synthesize crystals with controlled nanostructural characteristics and precise compositions.^63^

## Supporting Information Available

Further details and insights of the MD simulations carried out in this work including the atomistic representation of AuNP during the annealing treatment and distribution of mutual distances between the Au atoms as a function of time; MD snapshots regarding the adsorption process of 30, 20, and 10 PLGAs, respectively, at 0 and 100 ns, on the AuNP surface; distribution of mutual distances between the PLGA atoms as a function of time in the case studies: 30 PLGAs, 20 PLGAs, and 10PLGA; number of PLGA clusters formed during 100 ns of MD simulation in case of 30 initial PLGA molecules and 10 initial PLGA molecules; US histograms of one single simulation replica applied to the PLGA and AuNP in four distinct reciprocal orientations; convergence progress for the US method applied to the PLGA and AuNP at one reciprocal orientation; system-size independence study at 15.1 mM and 22.5 mM of PLGA concentration during the self-assembly on the AuNP surface; comparison of PLGA adsorption in case of polarizable and nonpolarizable Au force field; and comparison of adsorption mechanisms on the AuNP in case of deprotonated and protonated PLGAs (PDF)
Jupyter-Notebooks, Packmol code input files, and GROMACS software input files are shared via the public GitHub repository: https://github.com/robertacappabianca/SM_AuNP_PLGA.
