# Supplementary material for: Assembling Biocompatible Polymers on Gold Nanoparticles: Toward a Rational Design of Particle Shape by Molecular Dynamics
Source: ACS Omega. 2022 Nov 10;7(46):42292–303. doi: 10.1021/acsomega.2c05218 (PMC9686196; doi:10.1021/acsomega.2c05218)
Supplement: Supplementary file 1 — ao2c05218_si_001.pdf [file ao2c05218_si_001.pdf]

Supporting Information: Assembling Biocompatible Polymers on Gold Nanoparticles: Toward a Rational Design of Particle Shape by Molecular Dynamics

Roberta Cappabianca,[†,¶] Paolo De Angelis,[†,¶] Annalisa Cardellini,[*,†] Eliodoro Chiavazzo,[*,†] and Pietro Asinari[*,†,‡]

†*Department of Energy "Galileo Ferraris", Politecnico di Torino, Corso Duca degli Abruzzi 24, 10129 Torino, Italy*

‡*Istituto Nazionale di Ricerca Metrologica, 10135 Torino, Italy*

¶*Contributed equally to this work*

E-mail: annalisa.cardellini@polito.it; eliodoro.chiavazzo@polito.it; pietro.asinari@polito.it

Contents

1 **Annealing protocol to design a gold nanoparticle** **S-3**

2 **Molecular dynamics simulations and analyses** **S-5**

3 **System-size-independence study of PLGA adsorption** **S-10**

4 **Comparison of a polarizable and a nonpolarizable force field for gold atoms** **S-14**

5 **Comparison between deprotonated and protonated PLGAs adsorbing on the gold nanoparticle** **S-15**

6 **Summary of simulation efforts** **S-18**

References **S-19**

1 Annealing protocol to design a gold nanoparticle

In this section, we present a different method for designing a Gold Nanoparticle (AuNP). Referring to the work of Kyrychenko et al.,[S1] we consider an amorphous gold nanoparticle and we apply an annealing treatment to it. Specifically, such treatment consists first of heating the AuNP at high temperature and then of slowly cooling it to the room temperature. Through this method, we simulate the transition from a metastable condition to a stable condition. First, the Packmol code is used to place 1956 gold atoms inside a sphere with a radius of 2 nm in order to obtain the amorphous gold NP (i in figure S1a). Subsequently, the AuNP is placed in the center of a triclinic box, at a distance of at least 3 nm from the edge to ensure compliance with the minimum image convention. Solvation of the box is performed using water molecules described as the SPC/E water model.[S2] Periodic boundary conditions are applied in all directions. The AuNP is modeled by using the nonbonded parameters validated by Hendrik Heinz et al.[S3] Short-range interactions are evaluated within a cutoff radius of 1 nm, while a particle-mesh summation Eward (PME)[S4] is applied for long-range interactions. Finally, following our molecular dynamics (MD) protocol, we perform an energy minimization of the initial configuration and heat the resulting state to a temperature of 450 K. To heat the system we use a canonical ensemble (NVT) for 100 ps applying a Maxwell-Boltzmann speed distribution and V-rescale[S5] thermostat with a time constant τ_t=0.1 ps. During the equilibration step, the AuNP is constrained in its initial position using a harmonic potential with a force constant of $1000\,\mathrm{kJ\,mol^{-1}\,nm^{-2}}$. Once the system is heated, the constraint is removed and we leave the AuNP to freely move for 15 ns (ii in figure S1a). After that we extend the simulation for extra 8.4 ns in order to carry out slow cooling to 298 K. Specifically, we perform three cooling cycles starting at T=450 K and reducing the temperature by 10 K every 50 ps to take into account the relaxation time inherent in the coupling algorithm (iii in figure S1a). MD simulations integrate Newton's second law with the Leap-Frog algorithm[S5] and time step of 2 fs.

To show the evolution of the AuNP structure during the annealing, we analyze the relative

distribution of the atoms within the AuNP. Explicitly, we calculate the gold atomic distribution ($g^*_{AU/AU}(r)$), without normalizing it over the averaged gold atomic density (see figure S1b). In the first steps of annealing the $g^*_{AU/AU}(r)$ trend describes an amorphous structure, i.e without a well-defined configuration. However, during the simulation we notice the formation of ordered peaks mainly for r values smaller than about 0.5 nm. The structure of the AuNP from completely amorphous tends to form a crystalline core with variations in shape and surface coordination.

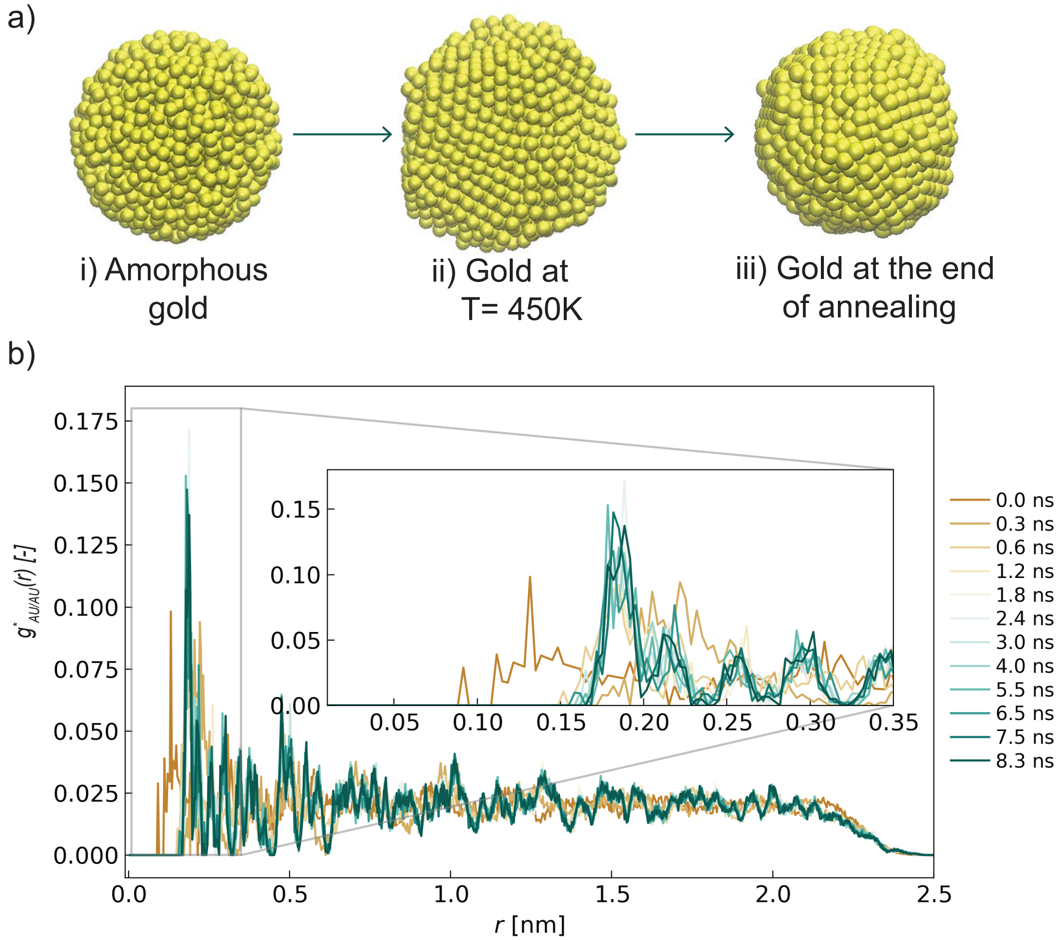

Figure S1: a) Atomistic representation of the AuNP during the annealing treatment: i) initial configuration of amorphous AuNP; ii) 450 K heated AuNP; iii) final configuration at the end of annealing. b) Distribution of mutual distances between the Au atoms ($g^*_{AU/AU}(r)$) as function of time; the zoom highlight the formation of crystalline structure in the AuNP core.

2 Molecular dynamics simulations and analyses

The procedure to define the atomistic model of crystalline AuNP is detailed in the Jupyter-Notebook interface available in the public GitHub repository. In the same repository there are the input files for the Packmol code and the GROMACS software. Specifically, the input files of the MD simulations, performed to investigate the self-assembly phenomenon of PLGAs on the AuNP in an aqueous solution are shared.

Figure S2 shows some representative snapshots of oligomers-NP self-assembly process in case of a single AuNP and (i) 30, (ii) 20, and (iii) 10 PLGAs, respectively at 0 ns and 100 ns. Moreover, in order to observe in a more quantitative way the progressive phenomenon of PLGA adsorption on the AuNP surface we display the relative distribution of atoms within the PLGA molecules ($g^{*}_{plga/plga}(r)$) in figure S3. The trend of $g^{*}_{plga/plga}(r)$ differs from the radial distribution function as it is not normalized over the averaged PLGA atomic density. Instead, in figure S4 we illustrate the clustering analysis results obtained for a single AuNP with 30 and 10 PLGAs, respectively. These results confirm that the self-assembly of the polymers is enhanced as the concentration increase, and it is faster than adsorption on the AuNP surface.

In the GitHub repository we have uploaded the Jupyter-Notebooks made for the post-processing analyses, namely i) clustering analysis and ii) water accessible surface. Finally, in the same repository there are the input files needed to use the umbrella sampling (US) method to calculate free energy (see table S1). Specifically, the US method allows sampling of all possible thermodynamic states along the reaction coordinate. Provided that some states are separated one each other by extremely high energy barriers (hills), the US approach facilitates the sampling of consecutive configurations by adding artificial harmonic potential and "flattening" those hills which otherwise would prevent the exploration of thermodynamics states when unbiased MD simulations are used. The sampling of the system is considered complete when it has "visited" the configuration space in each region defined along a reaction coordinate more than once. Therefore, the histogram of configurations must

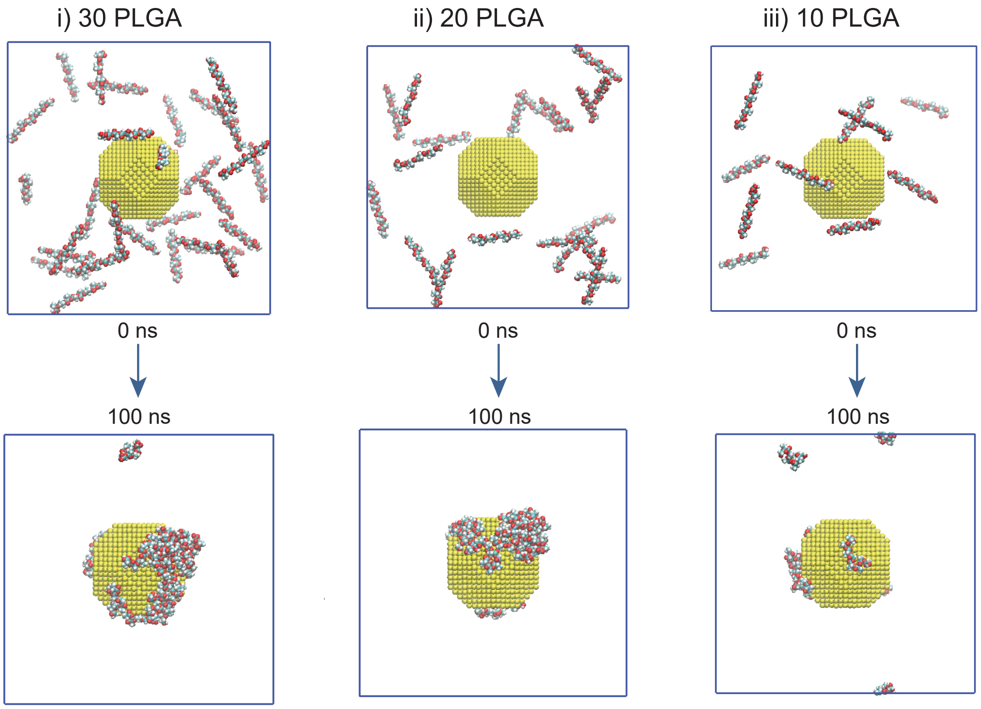

Figure S2: Molecular Dynamics snapshots of self-assembly simulations of the AuNP and i) 30, ii) 20 and iii) 10 PLGAs, respectively at 0 ns and 100 ns.

show a partial overlap between adjacent windows, in order to subsequently derive a continuous energy function from these simulations. MD simulations for different windows are run independently of each other, and then the weighted histogram analysis method (WHAM) is used to combine the statistics of all independent windows.[S6] Convergence of the method is improved by choosing appropriate sampling windows (see figure $S5$).

The convergence progress of US approach was also monitored by calculating the root mean square error (RMSE) between the potential of mean forces (PMFs) derived from the US simulations at different time instants. In figure $S6$, we show the convergence trend for one of the three simulations replica carried out for ξ perpendicular to the $\{1\ 1\ 1\}$ plane of the AuNP (case of $\theta = 54.7°$ see figure $S6a$). The RMSE of PMF becomes less than 1 kJ/mol after 9 ns (see figure $S6$c); therefore, we chose to use a simulation time for each sampling window of 10 ns to ensure the convergence of the results. In addition, in figure $S6$b we display the probability density of a single sampling window at different time instants in

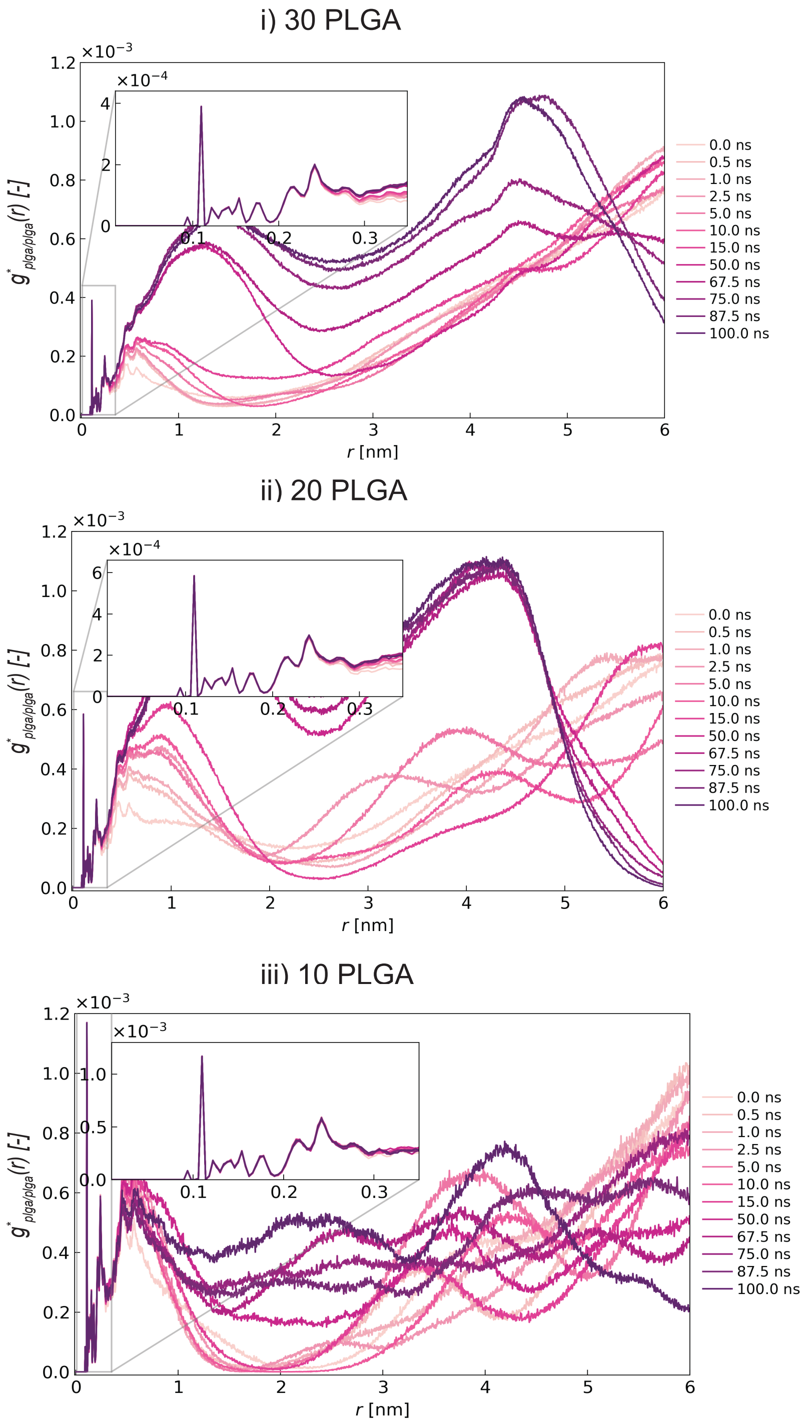

Figure S3: Distribution of mutual distances between the PLGA atoms ($g^*_{AU/AU}(r)$) as function of time for the case studies: i) 30 PLGAs; ii) 20 PLGAs; iii) 10 PLGA; the zooms display the atom distribution in a single polymer chain

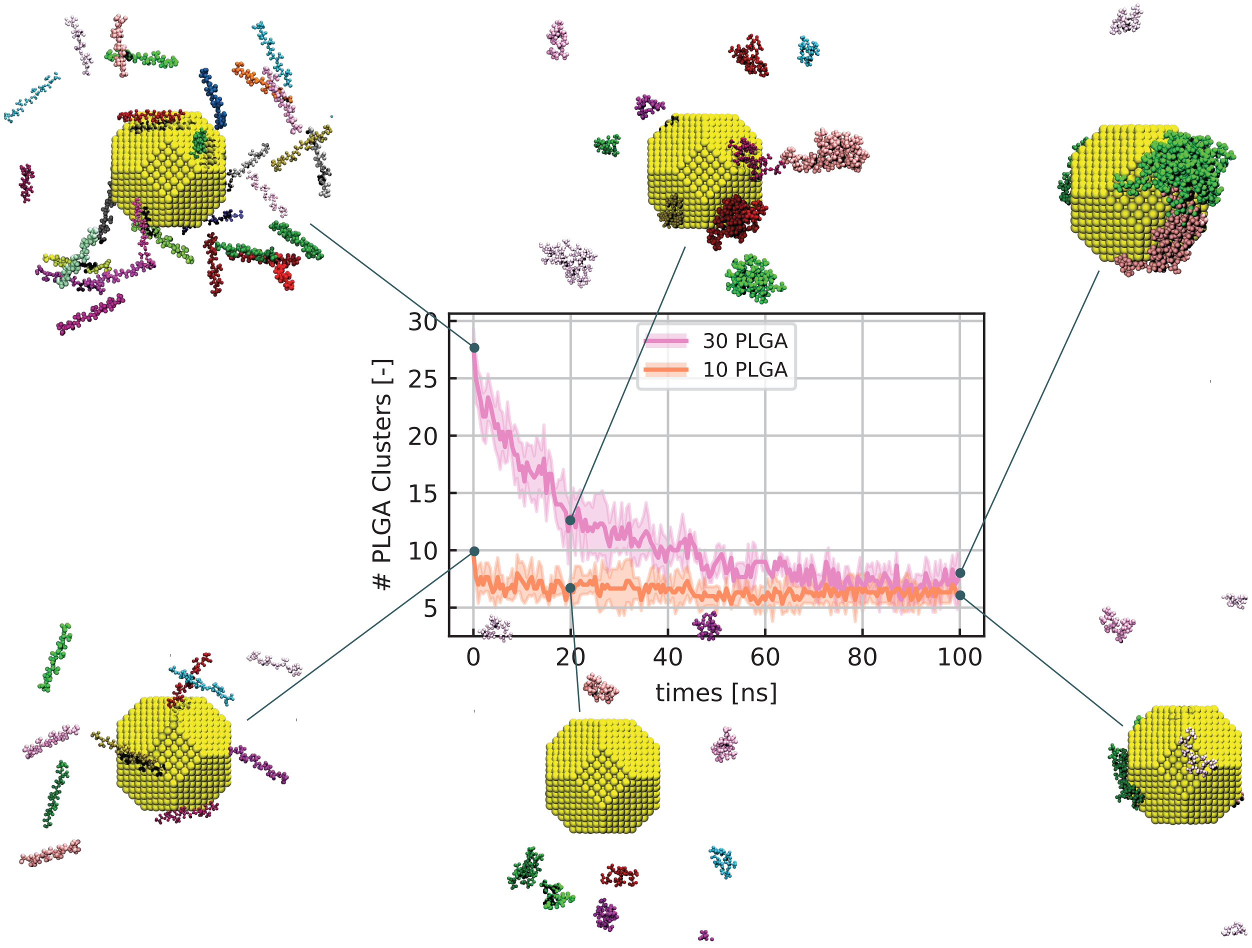

Figure S4: Number of PLGA clusters formed during 100 ns of MD simulation in case of 30 initial PLGA molecules (pink line, top snapshots) and 20 initial PLGA molecules (orange line, bottom snapshots). The color code used shows the atoms belonging to different clusters (note yellow is used to identify AuNP). The results are averaged over three replica copies of the same MD simulation and the shaded regions correspond to the standard deviation.

order to observe that by increasing the simulation time we approach to normal distribution that allows an ideal partial overlap between windows.

Table S1: Free energy of adsorption, Δ G, obtained by applying the US approach. The US protocol was repeated three times for each distinct reciprocal orientation between the AuNP and PLGA. Therefore, we averaged the results over the three replica copies and considered their mean and standard deviation.

**#PLGA chains**	**NP rotation angles**	**Δ G** [kJ/mol]	**#Replicas**
1	0°	187.85 ± 16.27	3
1	18.2°	176.72 ± 14.46	3
1	36.5°	176.93 ± 12.41	3
1	54.7°	212.02 ± 20.06	3

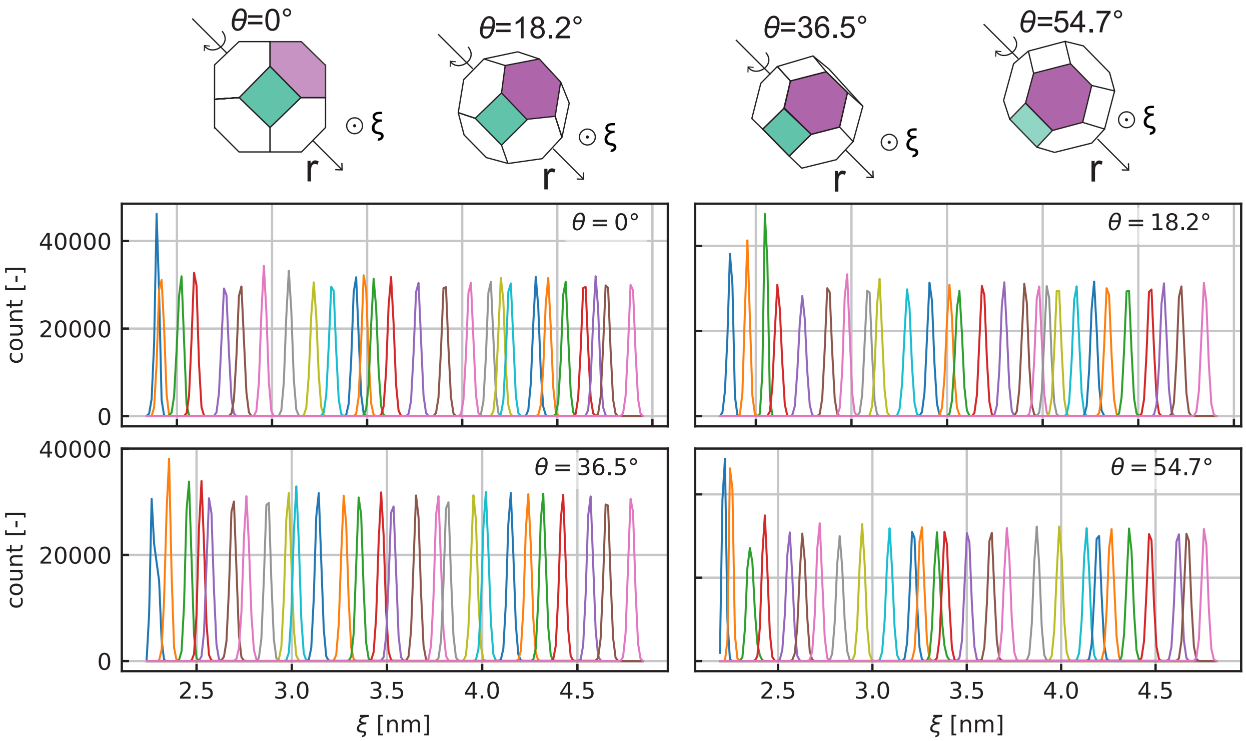

Figure S5: US histograms of one of three simulation replicas conducted with the US method applied to the PLGA and AuNP in four distinct reciprocal orientations. These four reciprocal configurations are realized by rotating the AuNP around the r-axis of θ.

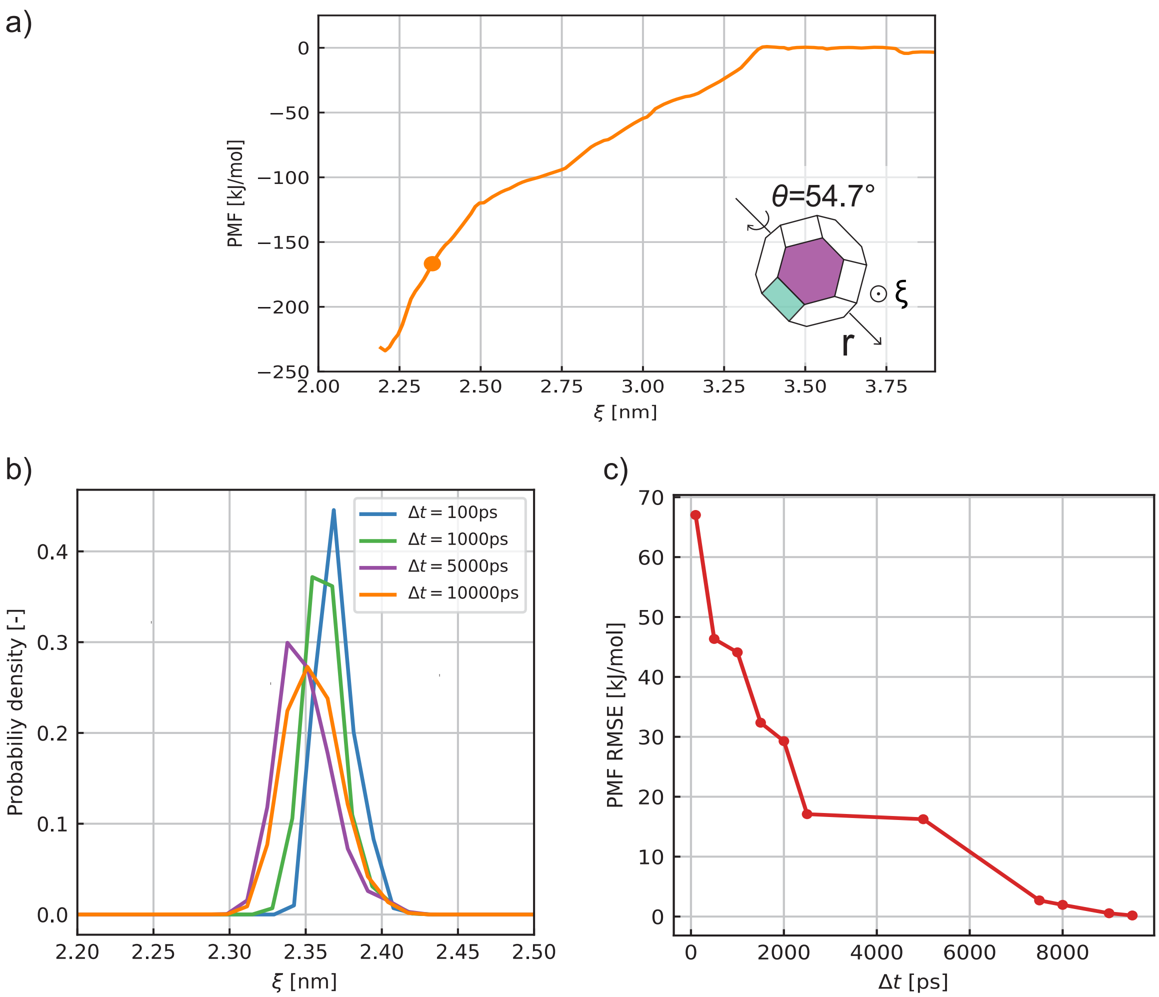

Figure S6: The convergence of the US method applied by pulling the PLGA from the AuNP surface to the bulk solution. The reciprocal orientation between PLGA and AuNP corresponds to $\theta = 54.7°$ a) The PMF is computed with a 10 ns of production run for each window. b) The time evolution of the probability density refers to the sampling window circled in panel a); the PMF value is extracted from the orange-colored distribution. c) The root mean square error (RMSE) between the PMFs at different time instants computed for the sampling configuration reported in panel a).

3 System-size-independence study of PLGA adsorption

In order to complete and validate our findings, we carried out a size-independent study of our system by increasing the box size of the simulations while keeping the molar concentration of PLGAs fixed at specific values. In particular, in figures S7 and S8 we considered a fixed PLGA concentration of 15.1 mM and 22.5 mM, respectively and we increased the box volume

from the initial 13x13x13 nm^3 by 23%, i.e., 16x16x16 nm^3, and by 54%, i.e., 20x20x20 nm^3. The results about PLGA clusters and SASA analysis show similar trends even considering a larger PLGA reservoir. The time evolution of PLGA clusters (figures S7a and S8a) confirms that first PLGA aggregates are formed and then their adsorption occurs on the surface of AuNP. We also evaluated the temporal evolution of SASA corresponding to the $\{1\,0\,0\}$ and $\{1\,1\,1\}$ crystalline planes and in relation to different box sizes (figures S7b and S8b). At initial time, the AuNP has 100% of its surface area in contact with water molecules. The percentage of SASA decreases more rapidly for $\{1\,1\,1\}$ crystalline planes than for $\{1\,0\,0\}$ planes, regardless the PLGA concentrations and box size. This validates our main result about the anisotropic adsorption: the PLGA polymer chains preferentially adsorb on the $\{1\,1\,1\}$ plane compared to the $\{1\,0\,0\}$ plane. Clear evidence is also demonstrated with the box size of 20x20x20 nm^3 (15.1 mM): after 100 ns while the $\{1\,1\,1\}$ uncoated area (i.e., the relative SASA) is roughly 60% of the initial area, the percentage of $\{1\,0\,0\}$ bare planes is still 80% of the total $\{1\,0\,0\}$ area. Moreover, for the same box size but higher concentration (22.5 mM) we obtain analogous findings: after 100 ns, while the $\{1\,1\,1\}$ uncoated area is a less than 40% of the initial area, the percentage of $\{1\,0\,0\}$ bare planes is still close to 60% of the total $\{1\,0\,0\}$ area. The histograms in figures S7c and S8c display the percentage of the total AuNP area still uncoated and highlight the proportion between the area $\{1\,1\,1\}$ (purple bars) and $\{1\,0\,0\}$ (cyan bars) exposed to water. All the results confirm the preferential adsorption of PLGA on the $\{1\,1\,1\}$ planes of the AuNP, regardless the system size and PLGA reservoir.

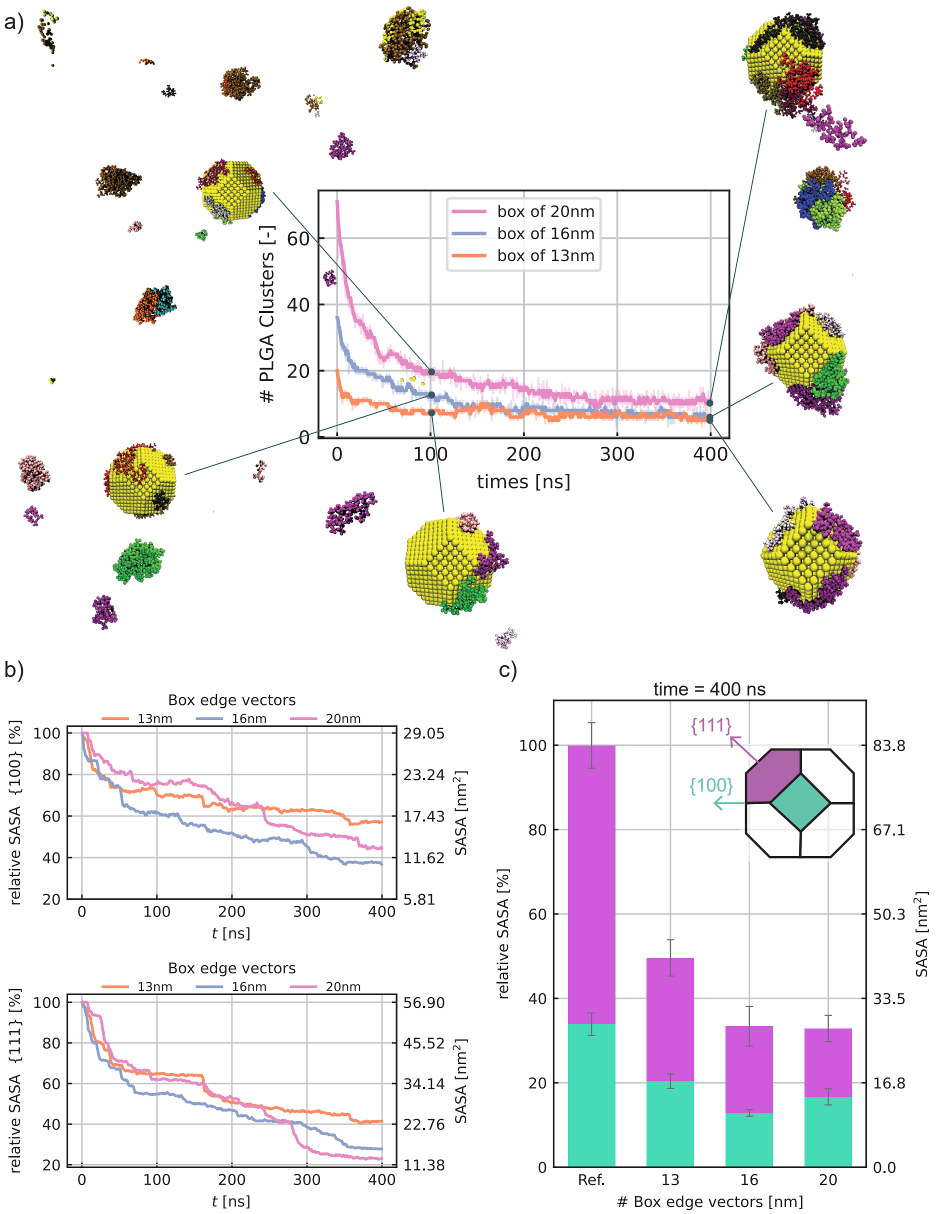

Figure S7: Self-assembly simulations of 15.1 mM PLGAs and one AuNP in aqueous solution for different box sizes: a) Number of PLGA clusters formed during 400ns in case of 13 nm (orange line, bottom snapshots), 16 nm (blu line, side snapshots) and 20 nm (pink line, top snapshots) box size; b) The SASA trends of {1 0 0} (top) and {1 1 1} (bottom) crystalline planes in the different box sizes (color legend as in a)). The relative SASA indicates the percentage values calculated taking the bare crystalline planes, while the right y-axes indicate the absolute value of the SASA; c) Histogram of the total AuNP SASA at 400 ns. Each bar is divided into two colors to distinguish the contribution of the two crystalline planes: purple color for the {1 1 1}, and cyan color for the {1 0 0}. The left y-axis is the percentage values of the relative SASA over the total AuNP surface area, while the right y-axis indicates the absolute value of the total AuNP surface. The 'Ref' bar indicates the reference surface area without PLGAs.

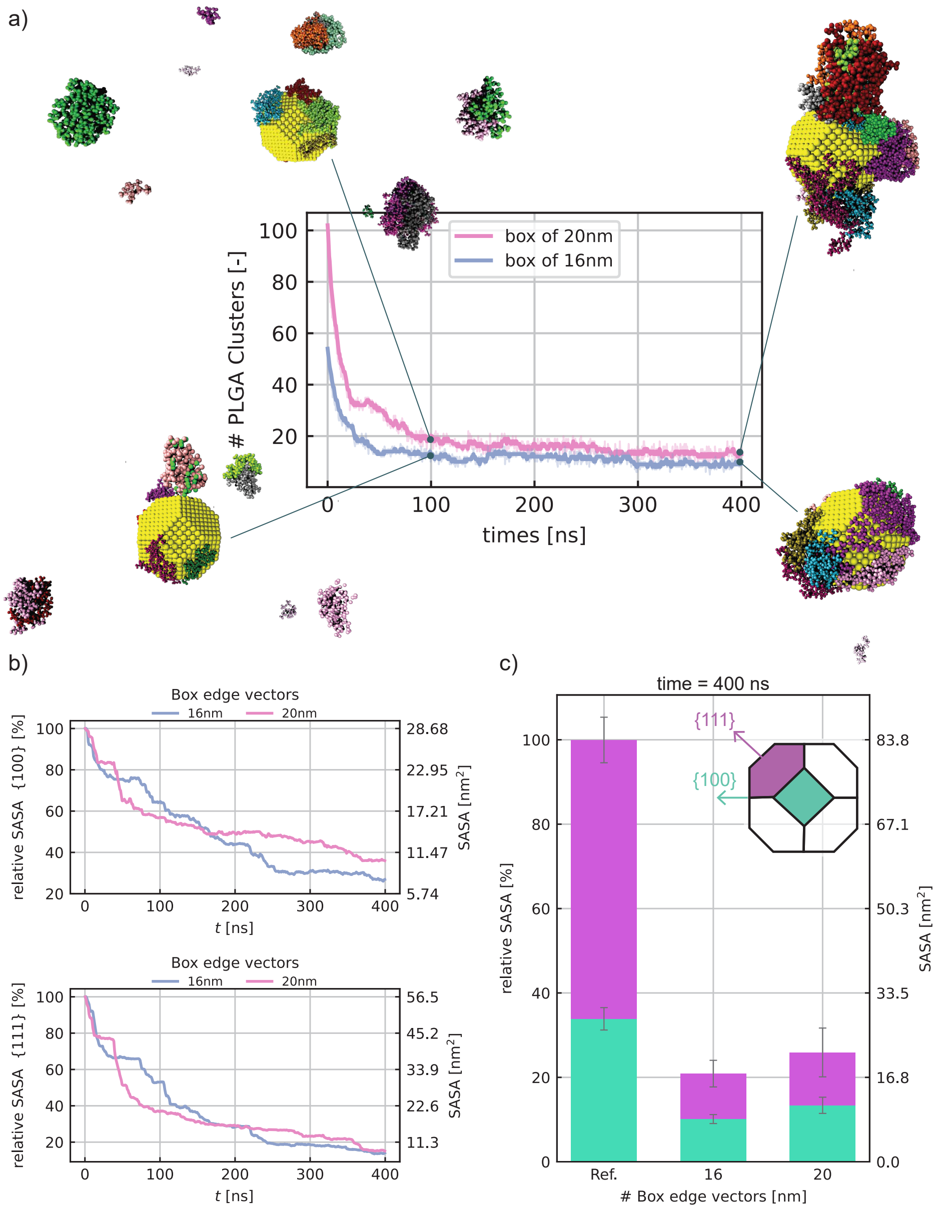

Figure S8: Self-assembly simulations of 22.5 mM PLGAs and AuNP in aqueous solution for different box sizes: a) Number of PLGA clusters formed during 400 ns in case of 16 nm (blu line, bottom snapshots) and 20 nm (pink line, top snapshots) box size; b) The SASA trends of {1 0 0} (top) and {1 1 1} (bottom) crystalline planes in the different box sizes (same color legend as a)). The relative SASA indicates the percentage values calculated taking the bare crystalline planes, while the right y-axes indicate the absolute value of the solvent accessible surface of the crystalline planes; c) Histogram of the total AuNP SASA at the last time step simulated, namely 400 ns. Each bar is divided into two colors to distinguish the contribution of the two crystalline planes: purple color for the {1 1 1} plane, and cyan color related to the {1 0 0} plane. The left y-axis is the percentage values of the relative SASA over the total AuNP surface area, while the right y-axis indicates the absolute value of the total AuNP surface. The 'Ref' bar indicates the reference surface area in the absence of PLGAs.

4 Comparison of a polarizable and a nonpolarizable force field for gold atoms

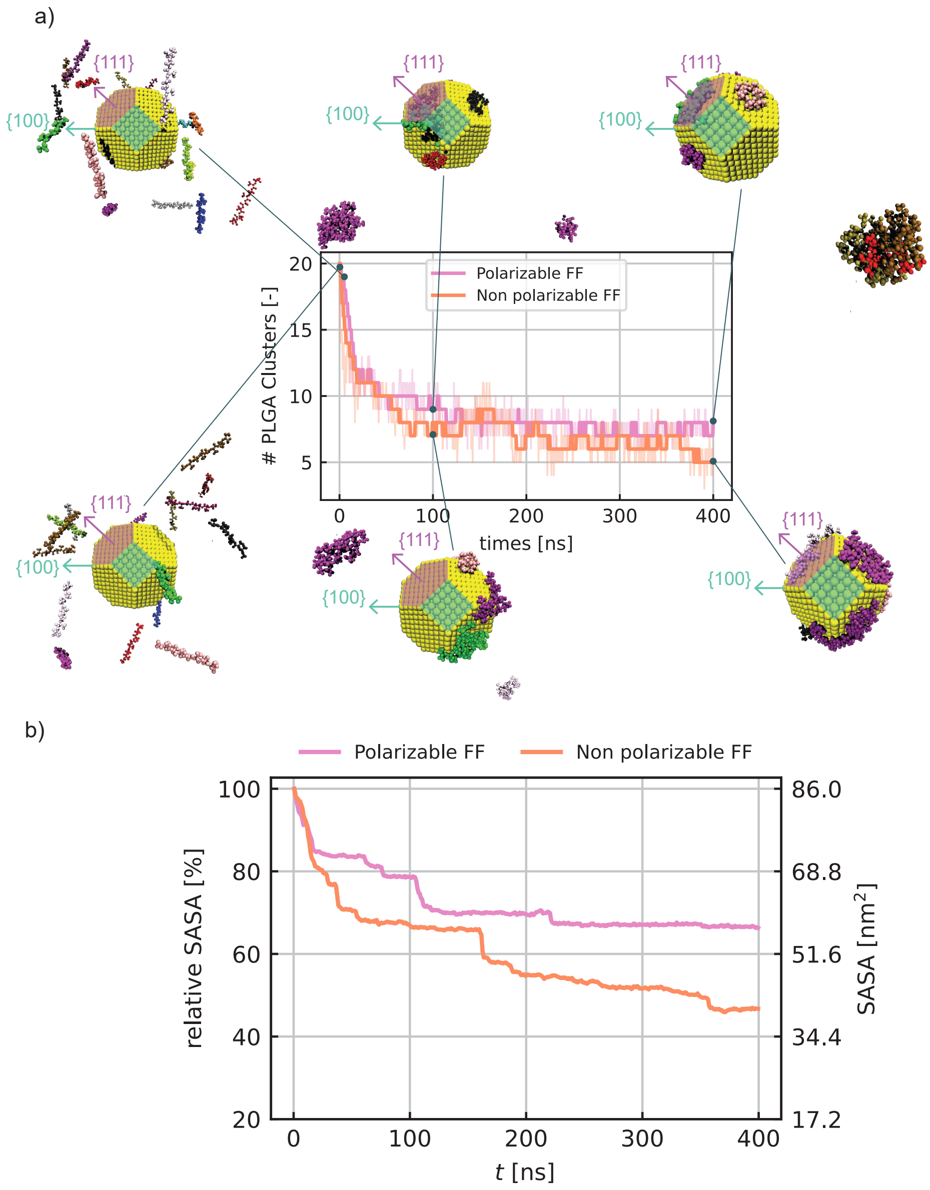

Figure S9: Self-assembly simulations of 15.1 mM PLGAs and AuNP in aqueous solution using two different force fields, with (pink) and without (orange) polarization of gold atoms. a) Number of PLGA clusters formed during 400 ns in case of using polarizable FF[S7] (pink line, top snapshots) and nonpolarizable FF (orange line, bottom snapshots). b) The SASA trends of total AuNP with the two different FF (same color legend as a)). The left y-axis is the percentage values of the relative SASA over the total AuNP surface area at the initial instant, while the right y-axis indicates the absolute value of the total AuNP surface.

5 Comparison between deprotonated and protonated PLGAs adsorbing on the gold nanoparticle

PLGA oligomer is characterized by an acid dissociation constant such that at room temperature in a physiological environment (neutral pH) tends to donate its protons. However, due to the presence of the pendant methyl group, deprotonated PLGAs exhibit predominantly hydrophobic behavior thereby self-assembling in aqueous environment. In the current work, we preliminary compared the behavior of protonated vs deprotonated PLGAs while simulating the adsorption phenomena on the AuNP surface. We performed 300 ns of self-assembly simulation of deprotonated PLGAs on the AuNP in aqueous solution. We considered a molecular modeling configuration including one AuNP and a concentration of deprotonated PLGAs of 45 mM in a box of 13x13x13 nm^3. Each deprotonated chain of PLGA consists of 67 atoms, after releasing two ester hydrogens. We followed the same MD protocol as described for protonated PLGAs. However, in this case PLGAs have a net charge of about -79e (based on its composition) so we added counterions to neutralize the system. As a result, the self-assembly of deprotonated oligomers occurs with salt bridges and not by forming acid-acid double h-bonds. The time evolution of PLGA clusters (figure S10a) confirms that the dynamics of the revealed adsorption mechanisms is the same: first the PLGA aggregates are formed and then their adsorption occurs on the AuNP surface. Figure S10a displays the cluster analysis in case of 60 deprotonated PLGA chains and 60 protonated PLGA chains in solution. Comparing the results, we observe that the deprotonation of PLGAs does not affect their self-assembly in clusters, which occurs mainly in the first 20 ns, however it tends to slow-down the PLGAs adsorption on the AuNP surface. To confirm the validity of our main result about the anisotropic adsorption, we also analysed the MD data with deprotonated PLGAs by monitoring the SASA per crystalline plane during the simulation (figure S10b). Thus, we evaluated the time evolution of SASA corresponding to the crystalline planes $\{1\ 0\ 0\}$ and $\{1\ 1\ 1\}$ in case of 60 deprotonated PLGA chains and 60 protonated PLGA chains. Along

the adsorption process, the percentage of the AuNP surface area exposed to water reduces more rapidly for {1 1 1} crystalline plane than for {1 0 0} in both tested systems. In the system with deprotonated PLGAs, the SASA of both crystalline planes is higher after 300ns. For example, the decrease of {1 1 1} SASA due to the protonated PLGAs adsorption is 92 %, while that due to the deprotonated PLGs adsorption is 64 %. This suggests that the different chemical structure of PLGAs makes them more stable in water, slowing down their adsorption onto the nanoparticle. As a matter of fact, the deprotonated state affects the velocity of aggregation mechanisms probably due to an enhanced hydrating effect of PLGAs. However, besides the velocity of adsorption, the main results regarding the adsorption are not affected: the favored adsorption site still remains the {1 1 1} crystalline plane. Indeed, in the system with deprotonated PLGAs after 300 ns while the {1 1 1} uncoated surface is the 36 % of the initial {1 1 1} area, the percentage of bare {1 0 0} planes corresponds to the 58 % of the total {1 0 0} area. The histograms in figure S10c display the percentage of the total AuNP area still uncoated after 300 ns and highlight the proportion between the area {1 1 1} (purple bars) and {1 0 0} (cyan bars) exposed to water. In case of 60 deprotonated PLGAs the percentage of bare AuNP surface after the adsorption is roughly the 45 % of the total AuNP area (see PLGAs in figure S10c). This uncoated surface presents a 55:45 ratio between the {1 1 1} and {1 0 0} planes, in contrast with the intrinsic plane surface ratio of the considered AuNP corresponding to 66:34 (see Ref. in figure S10c). The latter result is a clear demonstration that there is a preferential PLGAs adsorption on the {1 1 1} planes whether they are in the protonated or deprotonated state.

The comparison study between protonated and deprotonated PLGAs in solution confirms an analogous behavior of self-assembly and adsorption mechanisms on the AuNP surface, provided that protonated PLGAs are characterized of a faster dynamics. For this reason, in the current work, we considered MD simulations of protonated PLGA chains, which allowed us to reproduce the phenomena while saving computational time.

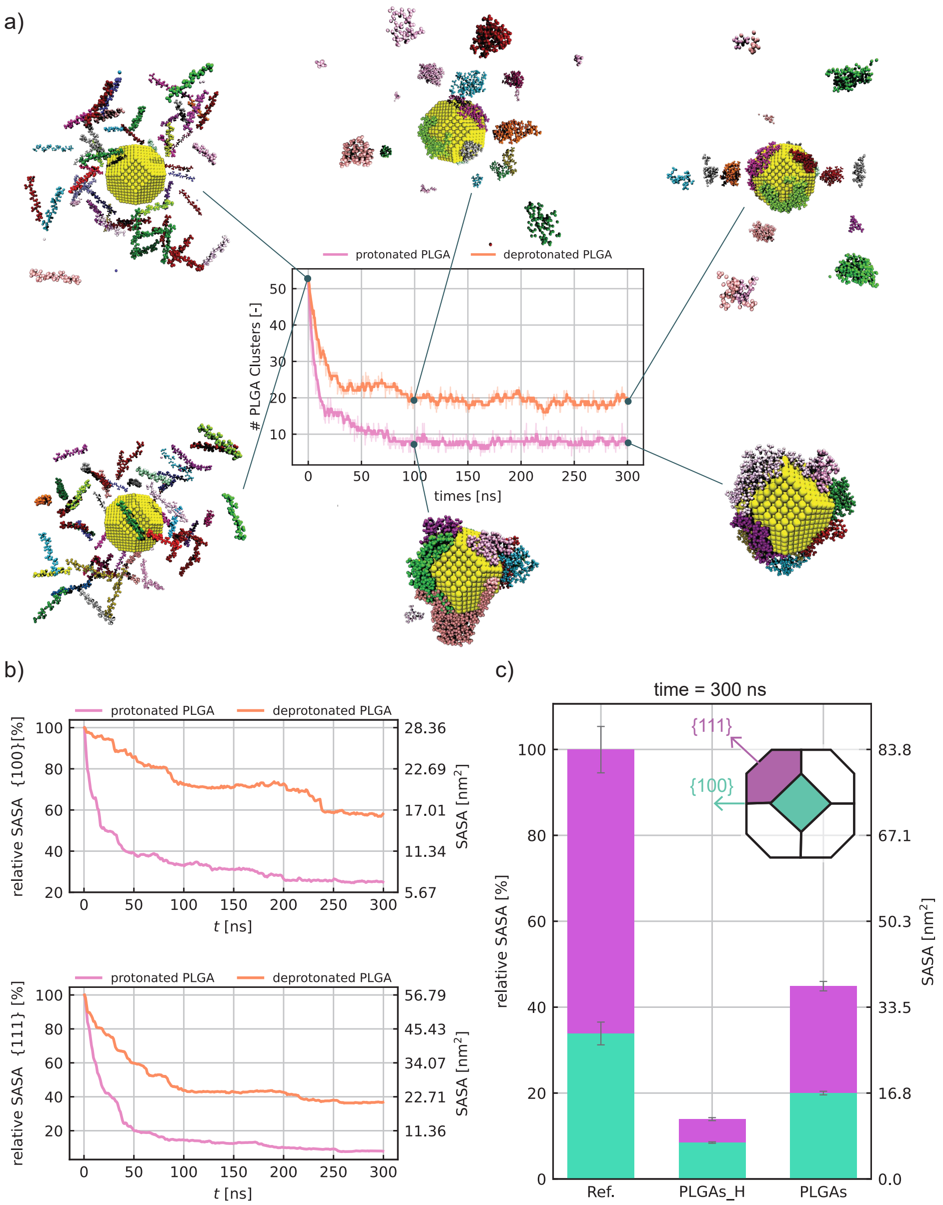

Figure S10: Comparison of adsorption mechanisms of 45 mM deprotonated and protonated PLGAs on the AuNP in aqueous solution. a) Number of PLGA clusters formed during 300 ns in case of protonated PLGAs (pink line, bottom snapshots) and deprotonated PLGAs (orange line, top snapshot). b) The time evolution of SASA of {1 0 0} (top) and {1 1 1} (bottom) crystalline planes due to the adsorption of PLGAs with different compositions (color legend as a)). c) Histogram of the total AuNP uncoated surface area at 300 ns; each bar is divided into two colors to distinguish the contribution of the two crystalline planes: purple color for the {1 1 1}, and cyan color for the {1 1 1}. On the x-axis, 'Ref' indicates the reference surface without PLGAs, 'PLGAs_H' is the surface with 60 protonated PLGAs, and 'PLGAs' represents the surface with 60 deprotonated PLGAs. Note that in both b) and c), the left y-axis is the percentage value of relative SASA over the bare crystalline planes and the total AuNP surface area, respectively, while the right y-axis indicates the absolute value of the SASA.

6 Summary of simulation efforts

Table S2: Self-assembly of PLGAs on AuNP in aqueous solution. Each PLGA chain has 69 atoms. Note that NPs with 2281 and 2171 Au atoms refer to the one with the {1 0 0} plane covering the 74% of the total surface area, and the one with the {1 1 1} plane covering the 78% of the total AuNP surface area, respectively.

**#Gold atoms**	**#PLGA chains**	**Box size** [nm^3]	**Time** [ns]	**#Replicas**
1925	0	13x13x13	100	1
1925	10	13x13x13	100	3
1925	20	13x13x13	100	3
1925	30	13x13x13	100	3
1925	60	13x13x13	100	3
2281	60	13x13x13	100	1
2171	60	13x13x13	100	1
1925	20	13x13x13	400	1
1925	37	16x16x16	400	1
1925	73	20x20x20	400	1
1925	56	16x16x16	400	1
1925	109	20x20x20	400	1

Table S3: US approach to calculate the PMF. The AuNP is rotated to calculate the PMF in different angular positions between the AuNP and the PLGA. We rotate the AuNP around the r-axis, orthogonal to both crystalline plane normal vectors and we selected configurations corresponding to four distinct θ rotation angles (see figure $S5$).

**#PLGA chains**	**NP rotation angles**	**Box size** [nm^3]	**Time** [ns]	**#Replicas**
1	0°	7x7x13	10	3
1	18.2°	7x7x13	10	3
1	36.5°	7x7x13	10	3
1	54.7°	7x7x13	10	3

References

(S1) Kyrychenko, A.; Karpushina, G. V.; Bogatyrenko, S. I.; Kryshtal, A. P.; Doroshenko, A. O. Preparation, structure, and a coarse-grained molecular dynamics model for dodecanethiol-stabilized gold nanoparticles. *Comput. Theor. Chem.* **2011**, *977*, 34–39.

(S2) Mark, P.; Nilsson, L. Structure and dynamics of the TIP3P, SPC, and SPC/E water models at 298 K. *J. Phys. Chem. A* **2001**, *105*, 9954–9960.

(S3) Heinz, H.; Vaia, R.; Farmer, B.; Naik, R. Accurate simulation of surfaces and interfaces of face-centered cubic metals using 12- 6 and 9- 6 Lennard-Jones potentials. *J. Phys. Chem. C* **2008**, *112*, 17281–17290.

(S4) Darden, T.; York, D.; Pedersen, L. Particle mesh Ewald: An N• log (N) method for Ewald sums in large systems. *J. Chem. Phys.* **1993**, *98*, 10089–10092.

(S5) Bussi, G.; Donadio, D.; Parrinello, M. Canonical sampling through velocity rescaling. *J. Chem. Phys.* **2007**, *126*, 014101.

(S6) Liao, Q. Enhanced sampling and free energy calculations for protein simulations. *Prog. Mol. Biol. Transl. Sci.* **2020**, *170*, 177–213.

(S7) Geada, I. L.; Ramezani-Dakhel, H.; Jamil, T.; Sulpizi, M.; Heinz, H. Insight into induced charges at metal surfaces and biointerfaces using a polarizable Lennard–Jones potential. *Nat. Commun.* **2018**, *9*, 1–14.
